# Phytotherapeutic potential of *Gossypium barbadense* L. root as an alternative therapy for colitis: a comprehensive review with network pharmacology insights

**DOI:** 10.1007/s10787-026-02337-9

**Published:** 2026-07-21

**Authors:** Amna Abou Bakr, Walaa K. Elabd, Said S. Moselhy, Rehab Abdel-Hameed

**Affiliations:** Biochemistry Department, Faculty of Science, Ain Shams University, Cairo, Egypt

**Keywords:** *Gossypium barbadense*, Flavonoids, Colitis, Inflammatory bowel disease, NF-κB, oxidative stress, Network pharmacology

## Abstract

Inflammatory bowel disease (IBD), particularly ulcerative colitis (UC), remains a major global health challenge characterized by chronic, relapsing inflammation of the colonic mucosa. Although conventional pharmacotherapies, including corticosteroids and mesalamine are widely used for disease management, their long-term efficacy is often limited by systemic adverse effects, treatment resistance and reduced patient responsiveness. Consequently, growing attention has been directed toward plant-derived bioactive compounds owing to their multi-target therapeutic potential and distinct structural mechanisms. This review highlights the pharmacological potential of *Gossypium barbadense* L. (Egyptian cotton) root extract as a potential phytotherapeutic candidate for the management of colitis. We summarize current evidence regarding the molecular mechanisms through which its major phytochemical constituents, particularly gossypol and related polyphenolic compounds that modulate inflammatory and oxidative stress pathways. The principal mechanisms include inhibition of NF-κB and COX-2 signaling, enhancement of endogenous antioxidant defense system, preservation of intestinal epithelial barrier integrity and restoration of immune homeostasis. In addition, the immunomodulatory and redox-regulating properties of *G. barbadense* phytoconstituents are discussed in the context of intestinal inflammation. Furthermore, this review integrates recent advances in network pharmacology to elucidate the multi-component, multi-target interactions underlying the therapeutic effects of *G. barbadense.* By consolidating current preclinical evidence and computational pharmacology approaches, this review highlights the potential of *G. barbadense* root as a prospective source of novel therapeutic agent for IBD while identifying key knowledge gaps that should be addressed before clinical translation.

**Graphical abstract:**

**Figure:**
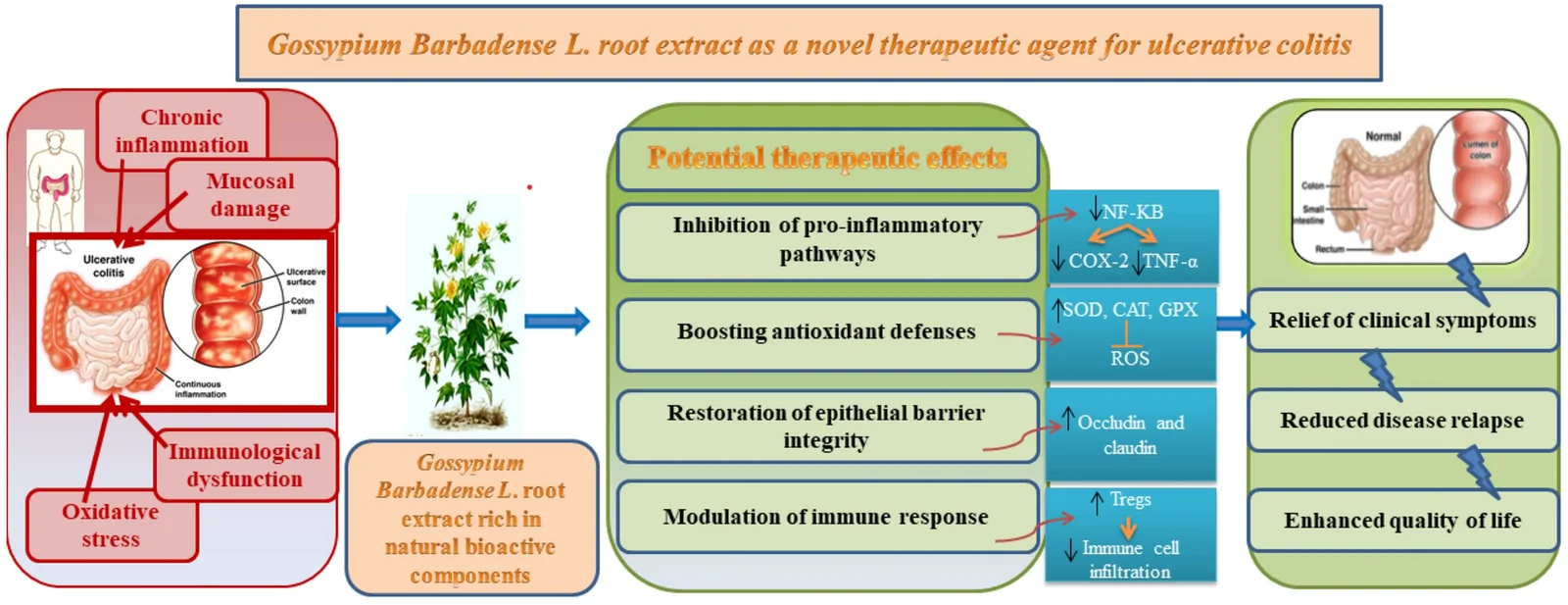

## Introduction

Colon plays a critical role in the absorption of water and electrolytes as well as in the processing of undigested food residues. Consequently, inflammation of the colon can disrupt normal bowel function, resulting in impaired nutrient absorption, systemic complications, and a substantial reduction in patients’ quality of life. Colitis is a chronic inflammatory condition of the colon associated with immune imbalance, oxidative stress, and progressive mucosal injury (Priyamvada et al. 2015). Although several pharmacological therapies are currently available, their long-term use is frequently associated with a significant side effects, incomplete remission, and disease recurrence. Therefore, increasing attention has been directed toward natural products and plant-derived bioactive compounds as a potential alternative or adjunctive therapeutic strategies due to their anti-inflammatory, antioxidant, and immunomodulatory properties (Abu Shelbayeh et al. 2023).

Colitis is a general medical term that refers to inflammation of the colon rather than a single disease entity. It encompasses several pathological conditions affecting the gastrointestinal tract (GIT) with distinct etiologies and pathological features. (Guandalini et al. 2015). Among the disorders, ulcerative colitis (UC) represents the most prevalent phenotype of inflammatory bowel disease (IBD). It is characterized by chronic inflammation and mucosal ulceration originating in the rectum and extending proximally through the colon, whereas Crohn’s disease (CD) may involve any region of the GIT (Alamgir 2018).

The global prevalence of IBD has escalated substantially over recent decades, imposing a significant clinical, social, and economic burden on healthcare systems worldwide (Alatab et al. 2020). Recent epidemiological data highlights this expanding burden, with documented cases affecting more than 4.9 million individuals globally. Although the highest prevalence remains in Europe and North America, the incidence of IBD is rising rapidly in developing regions including Asia, South America, and Africa (Heydari et al. 2025). The increasing disease burden has intensified the search for novel therapeutic agents capable of targeting multiple pathogenic mechanisms while minimizing adverse effects.

To date, diverse well-characterized natural products have demonstrated a robust ability to attenuate intestinal inflammation by modulating foundational protective pathways. Phytochemicals derived from *Curcuma longa* (curcumin), for instance, are widely recognized for suppressing canonical NF-κB activation (Gharge et al. 2021a), while *Boswellia serrata* extracts naturally inhibit 5-lipoxygenase (5-LOX) to reduce leukotriene-mediated mucosal injury (Ragab et al. 2024). Similarly, *Aloe vera* formulations have been shown to enhance tissue repair and upregulate tight junction proteins within the intestinal epithelium (Bahrami et al. 2020; Sánchez et al. 2020). Building upon these established botanical frameworks, the roots of *Gossypium species* offer a distinct, alternative therapeutic profile. They contain a unique combination of dimeric sesquiterpenoids (gossypol) alongside traditional flavonoids, tannins, and organic compounds, presenting a multi-target matrix that justifies focused pharmacological evaluation.

**Fig. 1:**
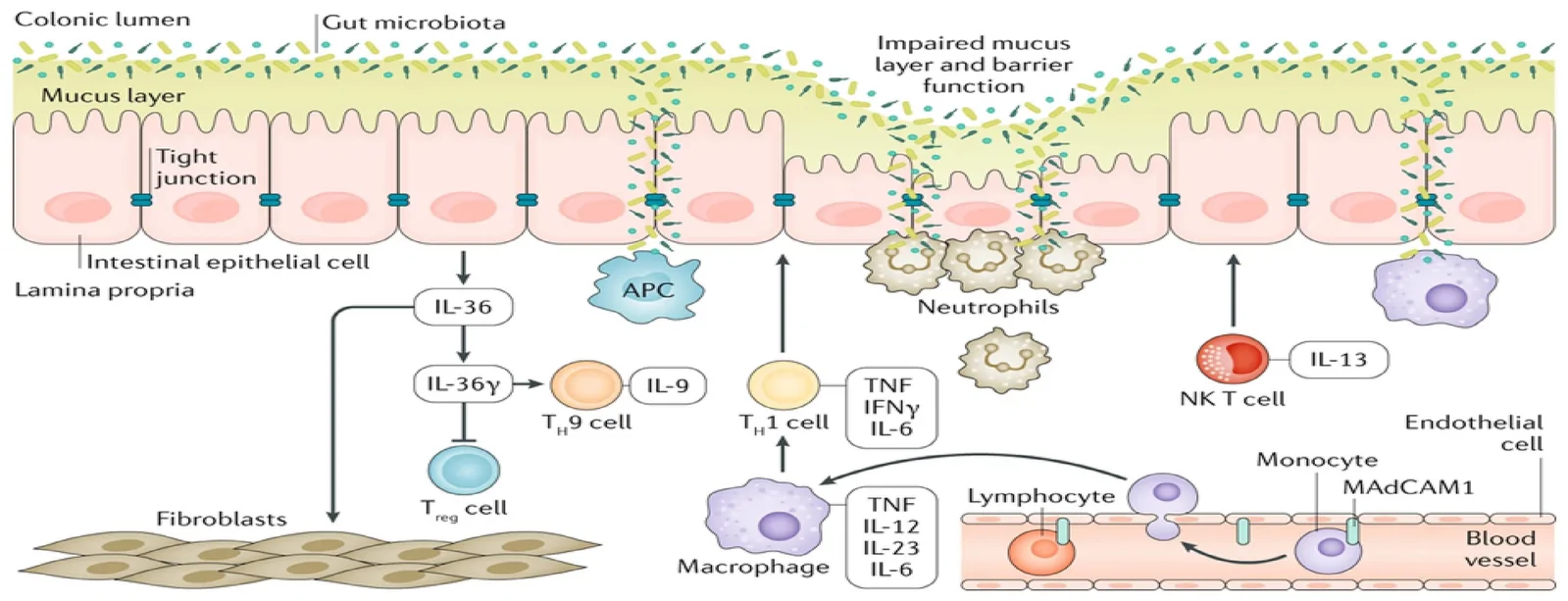
Inflammatory pathways causing colitis (Torres de Sousa Meneses 2023). Ulcerative colitis (UC) involves gut dysbiosis, reduced SCFAs, and impaired mucus production, leading to intestinal barrier disruption and microbial translocation. This triggers immune activation and release of pro-inflammatory cytokines (TNF-α, IL-6, IL-12, IL-23), promoting T cell responses and further barrier damage. Inflammation typically starts in the rectum and extends proximally. (IL, interleukin; TNF, tumor necrosis factor; IFN-γ, interferon gamma; APC, antigen-presenting cell; Th, T helper cells; NK T, natural killer T cells; MAdCAM-1, mucosal addressin cell adhesion molecule-1)

In ulcerative colitis (UC), chronic inflammation and progressive tissue damage are primarily driven by the excessive production of inflammatory mediators, including chemokines, cytokines, and proteases. These mediators promote the recruitment and activation of immune cells, sustain the inflammatory response, and compromise the integrity of intestinal mucosa. Patients with UC exhibit elevated levels of pro-inflammatory cytokines, particularly interleukin-1 β (IL-1β), interleukin-6 (IL-6), and tumor necrosis factor- α (TNF-α). Among these, TNF-α plays a pivotal role by promoting immune cell recruitment, trigger cytokine activation, inducing epithelial cell apoptosis, and disrupting tight junction proteins. Degradation of tight junctions and extracellular matrix components raises intestinal permeability, facilitating the translocation of luminal antigens and microorganisms into the underlying mucosa (Fig. 1). Furthermore, activation of key signaling pathways, including nuclear factor kappa B (NF-κB), Janus kinase signal transducer and activator of transcription (JAK/STAT), amplifies the production of pro-inflammatory mediators, thereby perpetuating intestinal inflammation and accelerating colonic tissue injury (Bu et al. 2025a).

Gut microbial dysbiosis also contributes to UC pathogenesis. Reduced abundance of beneficial commensal bacteria promotes immune polarization towards pro-inflammatory T helper (Th2 and Th17) response, leading to increased production of IL-13 and IL-17, and exacerbation of mucosal inflammation. In parallel, expansion of pathogenic bacteria taxa, particularly *Enterobacteriaceae* and *Proteobacteria phylum,* stimulate immune responses and drive inflammation through lipopolysaccharide secretion. The released lipopolysaccharide can stimulate Toll-like receptors (TLRs) on immune cells, triggering the production of cytokines and chemokines, boosting immune cell recruitment to the inflamed area, and prolonging inflammation and tissue injury. The resulting dysregulated immune response amplifies the inflammatory dysregulation in the gut, contributing to increased mucosal damage and inflammation. Overall, barrier impairment, immune cell infiltration, cytokine storms, and microbial imbalance create a self- amplifying inflammatory loop (Bu et al. 2025b).

Experimental studies have demonstrated that cotton-derived extracts containing gossypol, gossypin and related phytochemicals exhibit a significant anti-inflammatory and antioxidant activities through modulation of immune signaling pathways and suppression of inflammatory mediators. In addition, polyphenolic compounds such as flavonoids are widely recognized for targeting pathways like NF-κB signalling pathway, a central regulator of intestinal inflammation, thereby reducing cytokine production, oxidative stress, and immune cell activation (Alharbi et al. 2025).

These pharmacological properties are highly relevant to the pathogenesis of ulcerative colitis, in which chronic inflammation, oxidative stress, and epithelial barrier dysfunction are key contributors to disease progression. Polyphenolic compounds present in *Gossypium* root may attenuate these pathological processes by inhibiting the production of pro-inflammatory cytokines, restoring redox homeostasis, and preserving intestinal epithelial integrity, with NF-κB serving as one of the principal molecular targets (Al-Jashaami & Dupont 2016).

Recent advances in network pharmacology further support the therapeutic potential of *Gossypium* root by demonstrating that, its bioactive constituents can simultaneously interact with multiple molecular targets involved in intestinal inflammation. These targets include major inflammatory mediators such as TNF-α, IL-6, and IL-1β, as well as key regulatory pathways centered on NF-κB. Such multi-target interactions suggest that *Gossypium* phytochemicals modulate complex inflammatory networks rather than a single signaling pathway, providing a mechanistic basis for their use in the management of ulcerative colitis (Alsawak et al. 2023a, b).

Pathway enrichment analyses further indicated that, *Gossypium* root components modulate critical signaling cascades involved in the pathogenesis of ulcerative colitis, including NF-κB, MAPK, and PI3K/Akt pathways, which modulate inflammatory responses, oxidative stress, apoptosis, and intestinal epithelial barrier integrity. In addition, the key molecular targets such as PTGS2 (COX-2), NOS-2 (iNOS), and CASP-3 have been identified, highlighting their potential roles in modulating inflammation, nitric oxide production, and apoptosis regulation. Collectively, these findings support the therapeutic relevance of *Gossypium* root through a multi-component, multi-target, and multi-pathway mode of action (Amalraj et al. 2017).

**Fig. 2:**
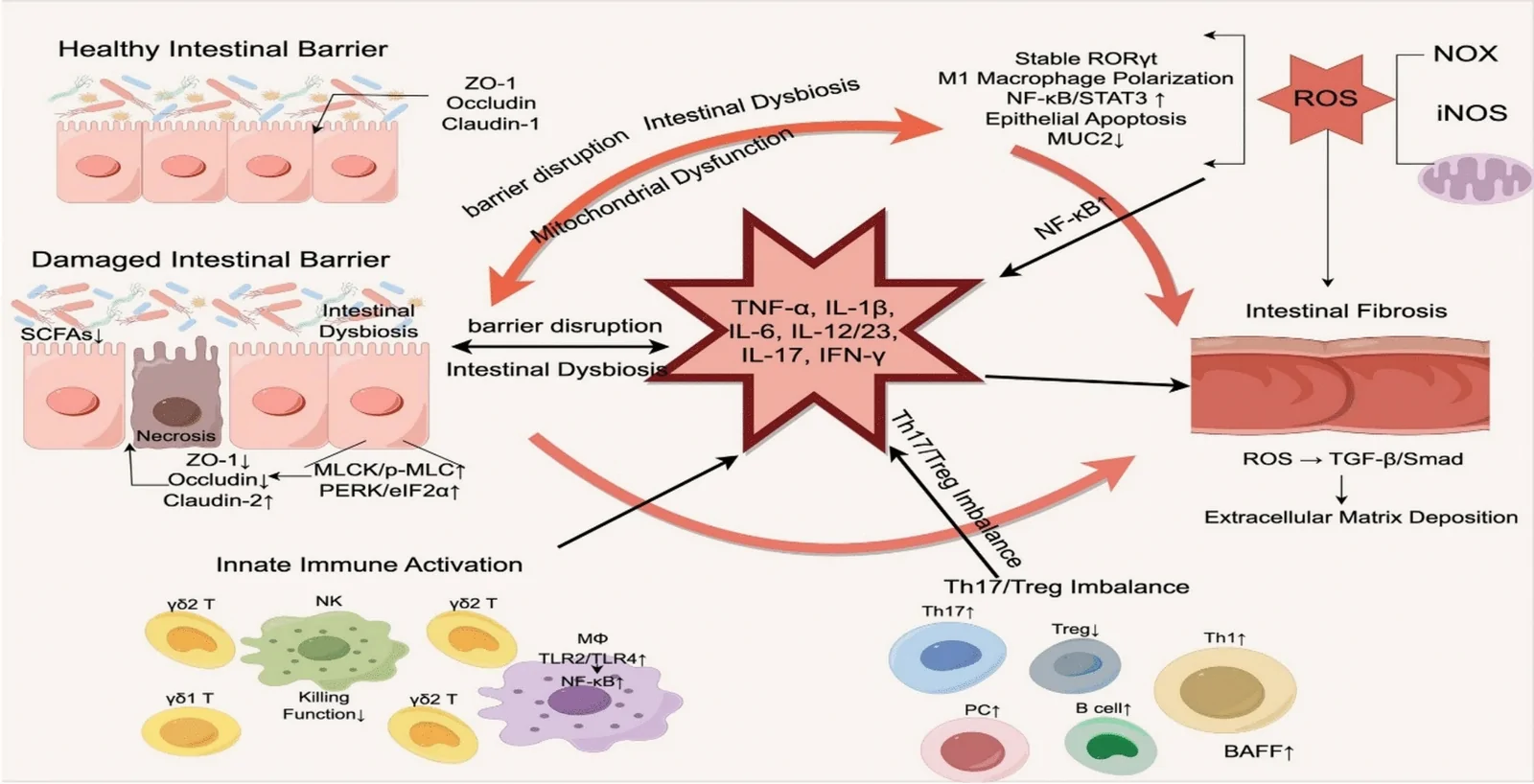
Oxidative stress pathway causing colitis (Yang et al. 2026). Intestinal dysbiosis and reduced SCFAs disrupt epithelial tight junctions, leading to barrier dysfunction, immune activation and ROS production via NOX and iNOS, activating NF-κB signaling and driving inflammation, Th17/Treg imbalance, and intestinal fibrosis. (ZO-1, Zonula Occludens-1; MLCK, Myosin Light Chain Kinase; p-MLC, phosphorylated Myosin Light Chain; PERK/eIF2α, Protein kinase R-like ER kinase / eukaryotic Initiation Factor 2α; NF-κB, Nuclear Factor kappa B; STAT3, Signal Transducer and Activator of Transcription 3; TGF-β/Smad, Transforming Growth Factor-beta / Mothers against decapentaplegic; NOX, NADPH Oxidase; iNOS, inducible Nitric Oxide Synthase; TNF-α, Tumor Necrosis Factor-alpha; IL-1β, IL-6, IL-12/23, IL-17, Interleukins; IFN-γ, Interferon-gamma; RORγt, RAR-related Orphan Receptor gamma t; Th17, T helper 17 cells; Treg, Regulatory T cells; NK, Natural Killer cells; γδ T, Gamma delta T cells; PC, Plasma Cells; TLR2/TLR4, Toll-like Receptors 2 & 4; MUC2, Mucin 2; SCFAs, Short-Chain Fatty Acids; ROS, Reactive Oxygen Species; BAFF, B-cell Activating Factor)

This review provides a comprehensive overview of the current understanding of ulcerative colitis pathophysiology and critically evaluates the phytochemical composition, pharmacological activities, and molecular mechanisms of *Gossypium barbadense L*. root. Particular emphasis is placed on evidence from experimental studies and recent network pharmacology analyses to elucidate its potential as a multi-target phototherapeutic candidate for the management of UC (Fig. 2).

## Materials

This review was prepared through a comprehensive literature search of major scientific databases, including PubMed, Scopus, Web of Science, and the National Institute of Health (NIH) database. Relevant peer-reviewed articles focusing on ulcerative colitis, inflammatory bowel disease, *Gossypium barbadense,* phytochemicals, oxidative stress, inflammatory signaling pathways, and network pharmacology were critically evaluated. Priority was given to recent studies and high-quality experimental and review articles that investigated the pharmacological activities and molecular mechanisms of *Gossypium barbadense* root constituents in the management of colitis.

### Etiology of colitis

The etiology of colitis is multifactorial and encompasses several distinct pathological conditions.

Infectious colitis is caused by bacterial pathogens such as *Shigella, Salmonella, Escherichia coli, Campylobacter jejuni, Mycobacterium tuberculosis,* and *Clostridium difficile*, as well as viral agents like cytomegalovirus (CMV) and parasites such as *Entamoeba histolytica* (Anwar et al. 2022a).

Ischemic colitis results from inadequate blood supply to the colon, leading to mucosal ischemia, ulceration, and gastrointestinal bleeding (FitzGerald and Hernandez 2015).

Radiation-induced colitis develops as a complication of pelvic radiotherapy administered for colorectal, urological, or gynecological malignancies (Arya et al. 2025).

**Fig. 3:**
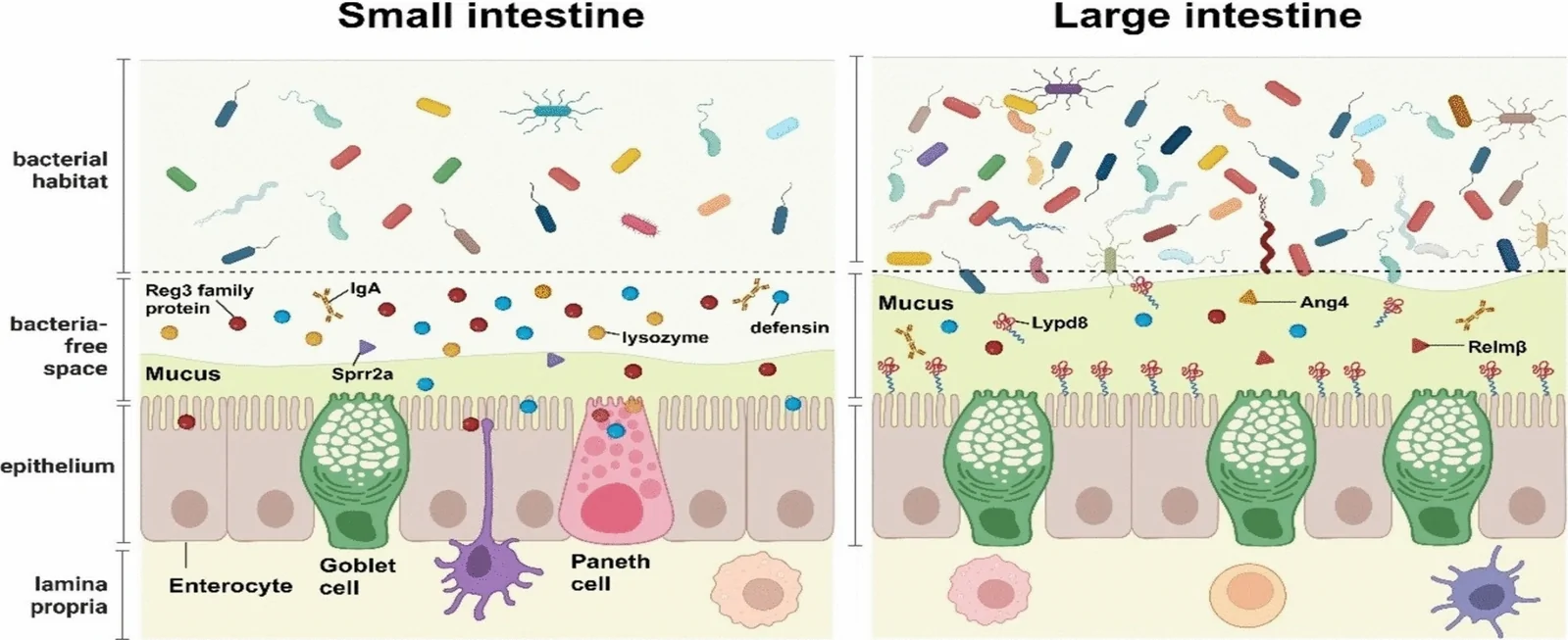
Gut barrier dysfunction leading to colitis (Okumura & Takeda 2025). From top to bottom: the *bacterial habitat* holds gut microbes, the *bacteria-free space* of mucus + antimicrobials block them, the *epithelium* of gut cells form the main physical barrier, and the *lamina propria* underneath provides immune defense

Microscopic colitis, which predominantly affects older adults, includes collagenous and lymphocytic colitis (Fig. 3). These conditions are diagnosed histologically and are frequently associated with autoimmune disorders. Including celiac disease, type-1 diabetes mellitus, thyroid disorders, and psoriasis (Bahrami et al. 2020).

Drug-induced colitis may occur following exposure to several medications, including aspirin, proton pump inhibitors, H₂-receptor antagonists, β-blockers, statins, immunosuppressive agents, non-steroidal anti-inflammatory drugs (NSAIDs), and vasopressors (Bai et al. 2023).

Recent advances in molecular biology and immunology have considerably improved our understanding of the complex interactions between genetic susceptibility, environmental factors, intestinal microbiota, and immune dysregulation in the pathogenesis of colitis. Nevertheless, the precise mechanisms underlying disease initiation and progression remain incompletely understood (Bakkaloğlu 2025).

### Pathogenesis and pathophysiology of colitis

Ulcerative colitis is a complex multifactorial disease resulting from interactions among genetic susceptibility, environmental factors, intestinal dysbiosis, epithelial barrier dysfunction, oxidative stress, and aberrant immune responses. These interconnected mechanisms collectively contribute to chronic intestinal inflammation, progressive mucosal injury and disease recurrence.

#### Inflammatory pathways

**Fig. 4:**
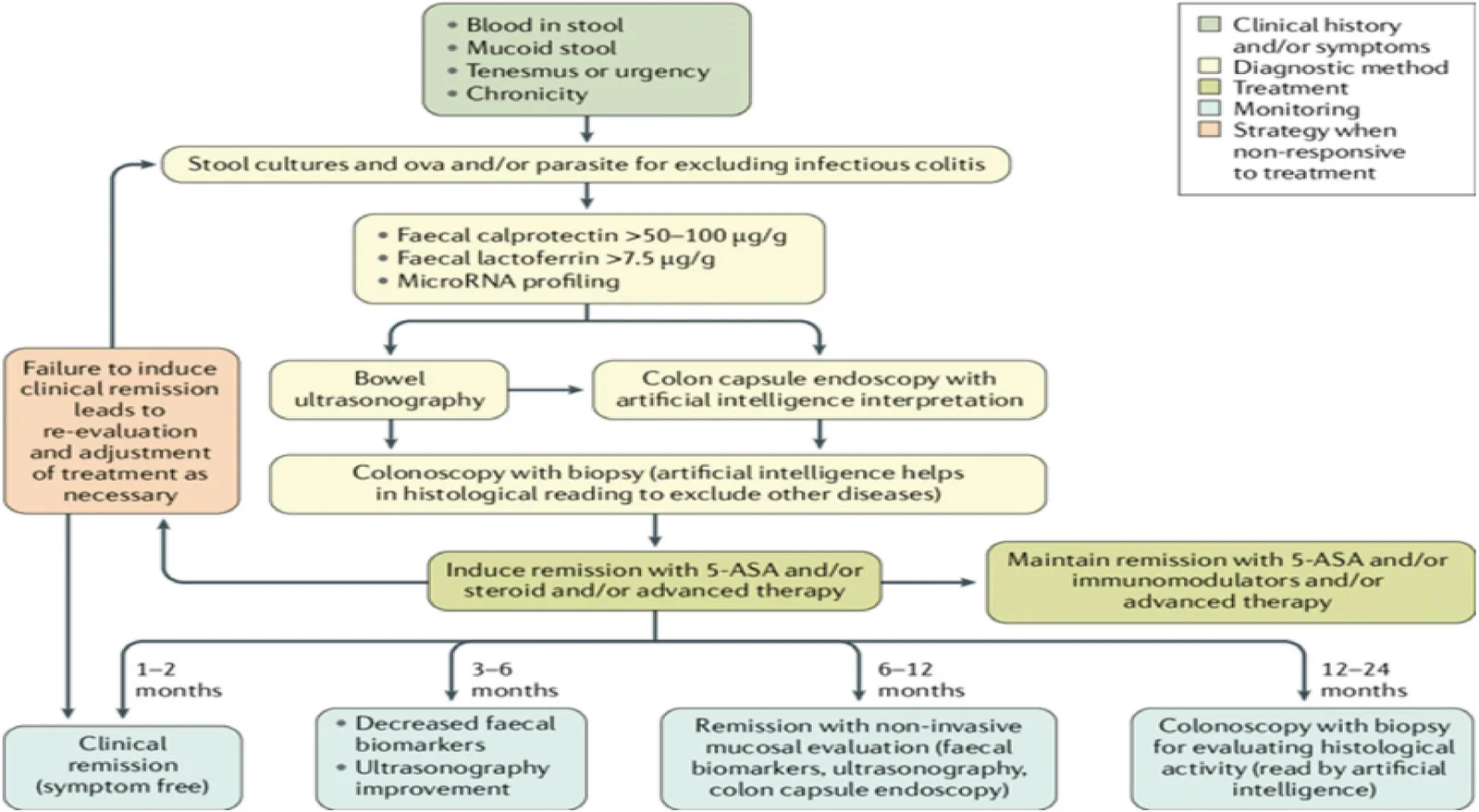
Diagnosis of colitis (Kobayashi et al. 2020a). Clinical presentation with blood, mucoid stool, and chronicity prompts exclusion of infectious colitis and assessment of fecal biomarkers. Remission is induced using 5-ASA, steroids, and/or advanced therapy, with treatment adjustment in non-responders. Maintenance therapy consists of 5-ASA, immunomodulators, and/or advanced therapy. Monitoring occurs at 1–2 months for clinical remission, 3–6 months for biomarker and ultrasonographic improvement, 6–12 months for non-invasive mucosal evaluation, and 12–24 months for colonoscopy with histological assessment. Box colors indicate clinical history/symptoms (dark green), diagnostic methods (beige), treatments (olive green), monitoring (light blue), and strategies for non-response (pink)

A hallmark of ulcerative colitis is dysbiosis, which is characterized by reduced microbial diversity and the depletion of beneficial bacteria that produce short-chain fatty acids (SCFAs). Reduced SCFA production impairs epithelial energy metabolism and weakens the intestinal mucus layer through the decreased synthesis of mucins, particularly MUC2 (Fig. 4). Consequently, disruption of the mucus barrier permits luminal microorganisms to directly interact with intestinal epithelial cells. Compromised epithelial integrity, together with the altered expression of tight junction proteins, increases intestinal permeability and facilitates bacterial translocation into the lamina propria. This process activates antigen-presenting cells (APCs) and macrophages, which subsequently produce chemokines that recruit neutrophils and additional immune cells to the site of inflammation (Murphy et al. 2022).

Activated neutrophils release neutrophil extracellular traps (NETs), whereas infiltrating macrophages produce high levels of pro-inflammatory cytokines, including TNF-α, IL-6, IL-12, and IL-23, thereby promoting T-helper cell polarization and amplifying the inflammatory response. Furthermore, epithelial-derived IL-36γ suppresses regulatory T cells (Tregs), promotes the differentiation of IL-9-producing T-helper (Th9) cells, and stimulates profibrotic signaling pathways. Natural killer T (NKT) cells also contribute to disease progression through IL-13 production, which further compromises epithelial barrier integrity (Chen and Boskovic 2024).

**Fig. 5:**
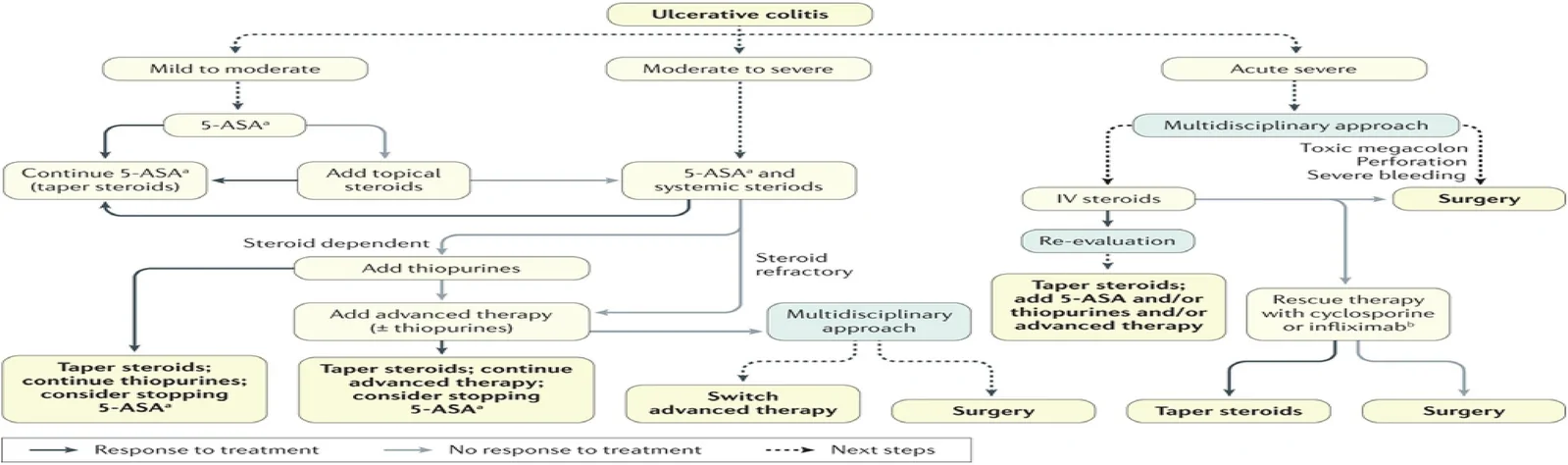
Different colitis conventional therapeutic methods (Kobayashi et al. 2020b). Management is stratified into mild-to-moderate, moderate-to-severe, and acute severe disease. Mild-to-moderate cases are treated with 5-ASA ± topical steroids, with steroid tapering on response. Moderate-to-severe cases use 5-ASA plus systemic steroids, progressing to thiopurines or advanced therapy in steroid-dependent or refractory disease. Acute severe colitis requires IV steroids and multidisciplinary review, with rescue therapy using cyclosporine or infliximab or surgery for non-response or complications. Solid arrows indicate treatment response, dashed arrows indicate non-response, and dotted arrows indicate next steps

These localized cytokine cascades activate overarching upstream signaling networks, specifically the mitogen-activated protein kinase (MAPK) and Janus kinase/signal transducer and activator of transcription (JAK/STAT) pathways (Fig. 5). These pathways systematically converge onto the nuclear factor-kappa B (NF-κB) transcription factor complex. Upon activation by upstream signals, the companion IκB kinase (IKK) complex phosphorylates downstream inhibitory proteins, facilitating the nuclear translocation of active p50/p65 heterodimers to drive chronic mucosal transcription cascades (Khasanov et al. 2026) In ulcerative colitis, inflammation typically begins in the rectum and extends proximally in a continuous pattern, resulting in the chronic activation of inflammatory signaling pathways and persistent mucosal injury (Murphy et al. 2022).

#### Oxidative stress pathways

Oxidative stress plays a pivotal role in both the initiation and progression of ulcerative colitis. Excessive production of reactive oxygen species (ROS) and reactive nitrogen species (RNS) by activated neutrophils and macrophages overwhelms endogenous antioxidant defense systems, resulting in oxidative damage to DNA, proteins, lipids, and cellular organelles (Balmus et al. 2016).

**Fig. 6:**
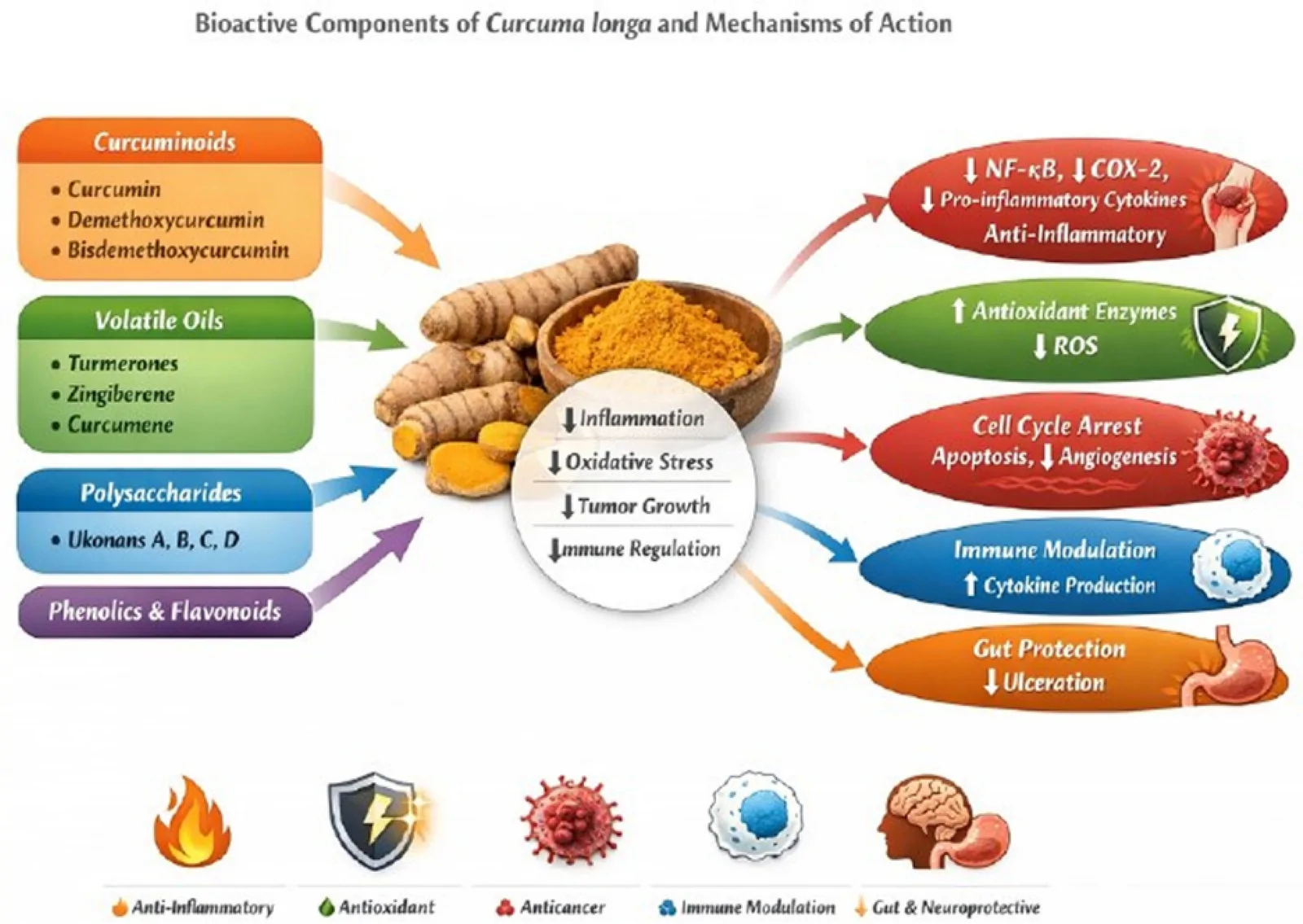
Pathways and effects of natural components of *Curcuma* on colitis (Gharge et al. 2021a, b). Curcuminoids, volatile oils, polysaccharides, and phenolics/flavonoids from turmeric exert anti-inflammatory, antioxidant, anticancer, immune-modulating, and gastroprotective effects. These actions occur through inhibition of NF-κB and COX-2, enhancement of antioxidant enzymes, induction of apoptosis, and modulation of immune and gut functions, leading to reduced inflammation, oxidative stress, and tumor growth

Elevated ROS levels activate multiple pro-inflammatory signaling cascades, including the NF-κB, MAPK, and STAT3 pathways, thereby promoting the transcription and translation of effector cytokines such as TNF-α, IL-1β, and IL-6. Simultaneously, oxidative stress disrupts tight junction proteins, increases intestinal permeability, and perpetuates mucosal inflammation (Fig. 6), creating a self-amplifying cycle of oxidative injury and chronic inflammation (Yang et al. 2026).

**Fig. 7:**
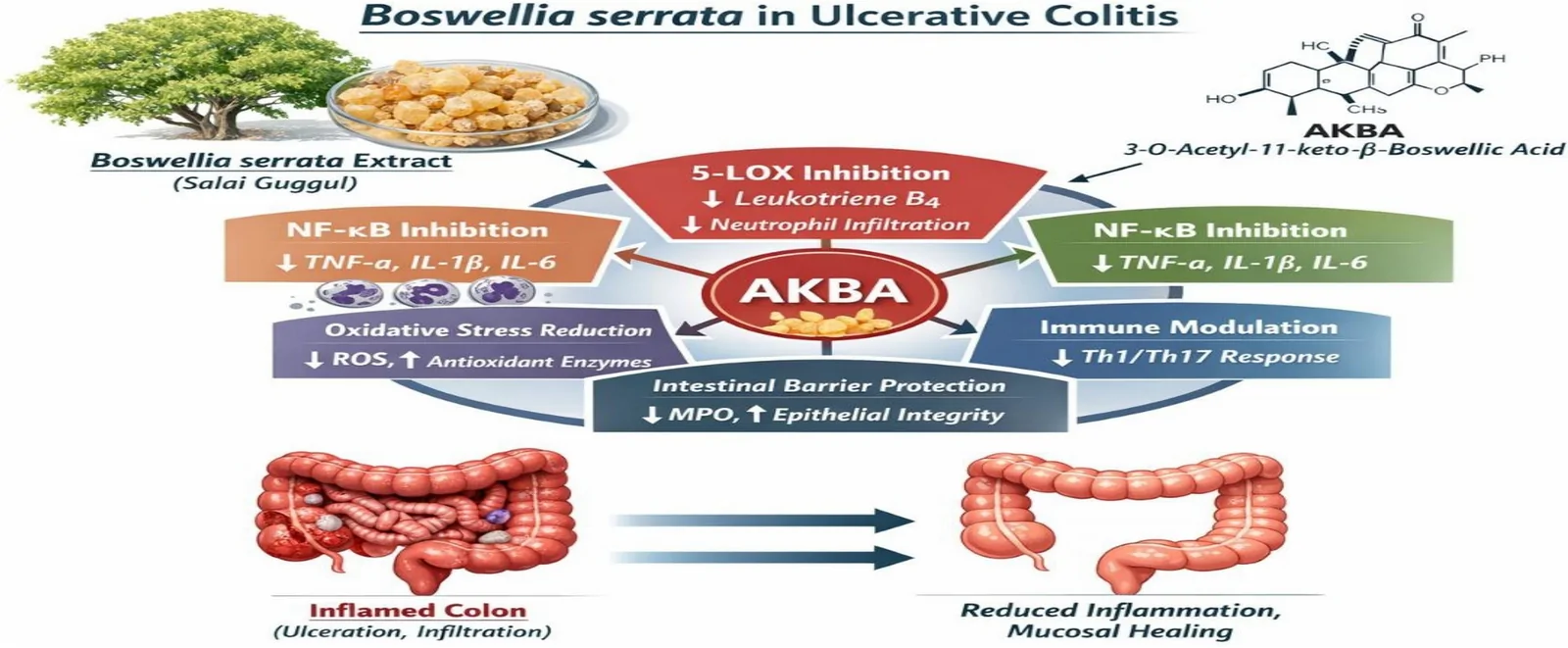
Pathways and effects of natural components of *Boswellia serrata* on colitis (Ragab et al. 2024). The active compound AKBA from _*Boswellia serrata*_ extract reduces colonic inflammation by inhibiting 5-LOX and NF-κB pathways, thereby decreasing leukotriene B4, neutrophil infiltration, and pro-inflammatory cytokines TNF-α, IL-1β, and IL-6. It also alleviates oxidative stress, modulates Th1/Th17 immune responses, and enhances intestinal barrier integrity. These effects transition the colon from an inflamed, ulcerated state to reduced inflammation and mucosal healing

Clinical studies have consistently demonstrated elevated oxidative stress biomarkers, including malondialdehyde (MDA) and 8-hydroxy-2′-deoxyguanosine (8-OHdG) (Fig. 7), in patients with active ulcerative colitis. These biomarkers correlate with disease severity, further supporting the central role of oxidative stress in disease pathogenesis (Matar et al. 2024).

#### Intestinal barrier dysfunction.

The intestinal epithelial barrier represents the first line of defense against luminal microorganisms and environmental antigens. In ulcerative colitis, pathological inflammation compromises epithelial tight junctions, which increases intestinal permeability and permits luminal bacteria, endotoxins, and dietary antigens to penetrate the mucosa and activate resident immune cells (Escalante et al. 2025; Arya et al. 2025).

This abnormal immune activation triggers sustained production of pro-inflammatory cytokines, including TNF-α and IL-6, which further impair tight junction integrity and perpetuate epithelial damage. Barrier dysfunction is closely associated with gut dysbiosis, creating a vicious cycle in which microbial imbalance promotes inflammation while inflammation further disrupts the epithelial barrier (Özsoy et al. 2022).

**Fig. 8:**
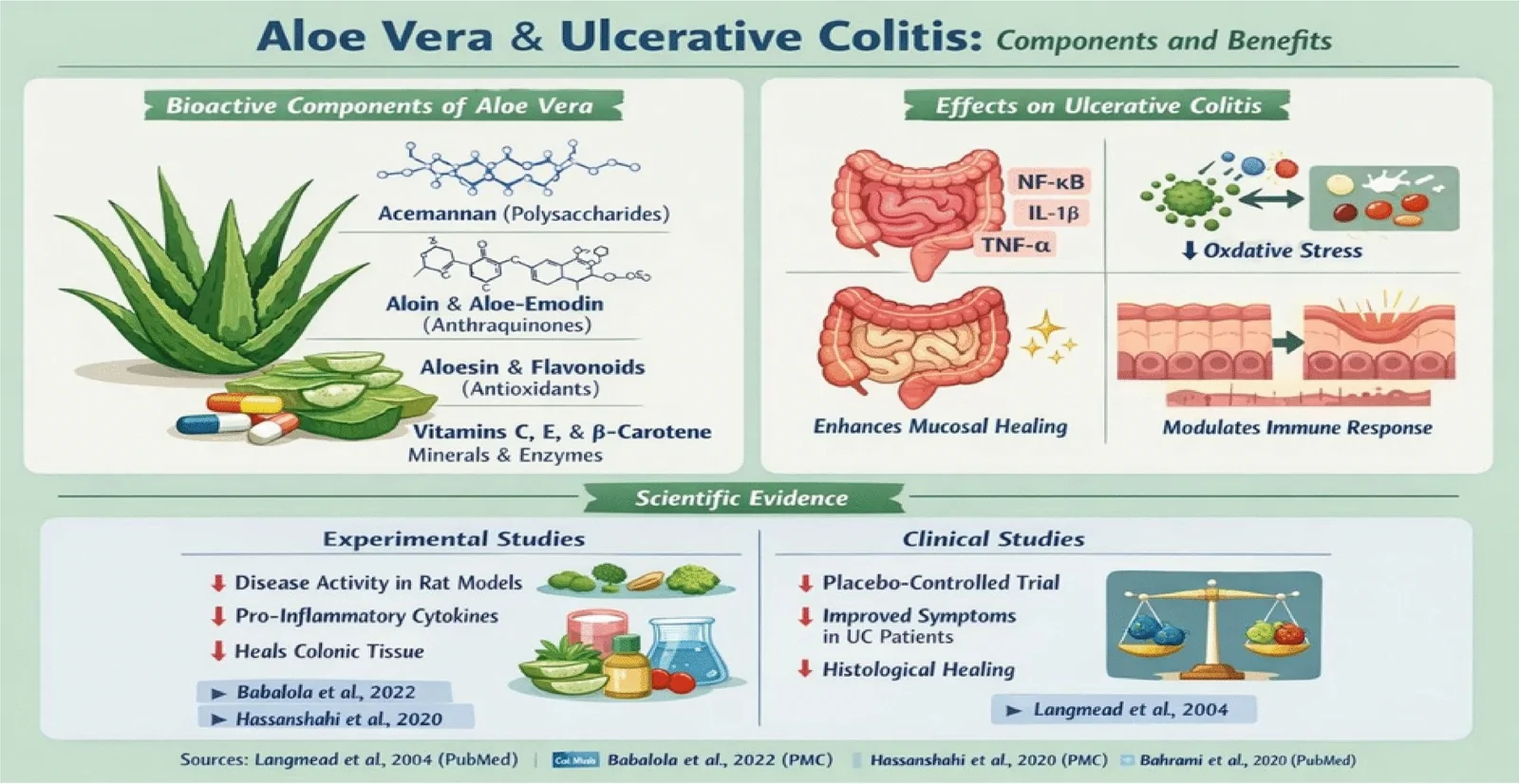
Pathways and effects of natural components of *Aleo vera* on colitis (Bahrami et al. 2020a) (Sánchez et al. 2020). In animal models of chemically induced colitis, particularly acetic acid– and DSS-induced colitis models, treatment with gel derived from *Aloe vera* has demonstrated significant therapeutic effects. Administration of *Aloe vera* gel was associated with a reduction in the disease activity index, and attenuation of colon shortening. Additionally, histological examination revealed improved mucosal architecture and reduced inflammatory cell infiltration, indicating enhanced tissue repair and protection against colonic damage. These findings collectively suggest that *Aloe vera* exerts anti-inflammatory and mucosal healing effects, supporting its potential as a complementary therapeutic agent for UC in experimental settings (Bahrami et al. 2020a)

Because intestinal epithelial cells rely heavily on mitochondrial oxidative phosphorylation for ATP production, mitochondrial dysfunction further compromises barrier integrity by reducing cellular energy availability and increasing susceptibility to oxidative injury (Fig. 8).

#### Mitochondrial dysfunction

Mitochondrial dysfunction has emerged as a critical contributor to ulcerative colitis pathogenesis. Damaged mitochondria generate excessive mitochondrial reactive oxygen species (mROS), which activate inflammatory signaling pathways, increase pro-inflammatory cytokines, induce epithelial apoptosis, and impair intestinal barrier function (Wang and Xiong 2025).

**Fig. 9:**
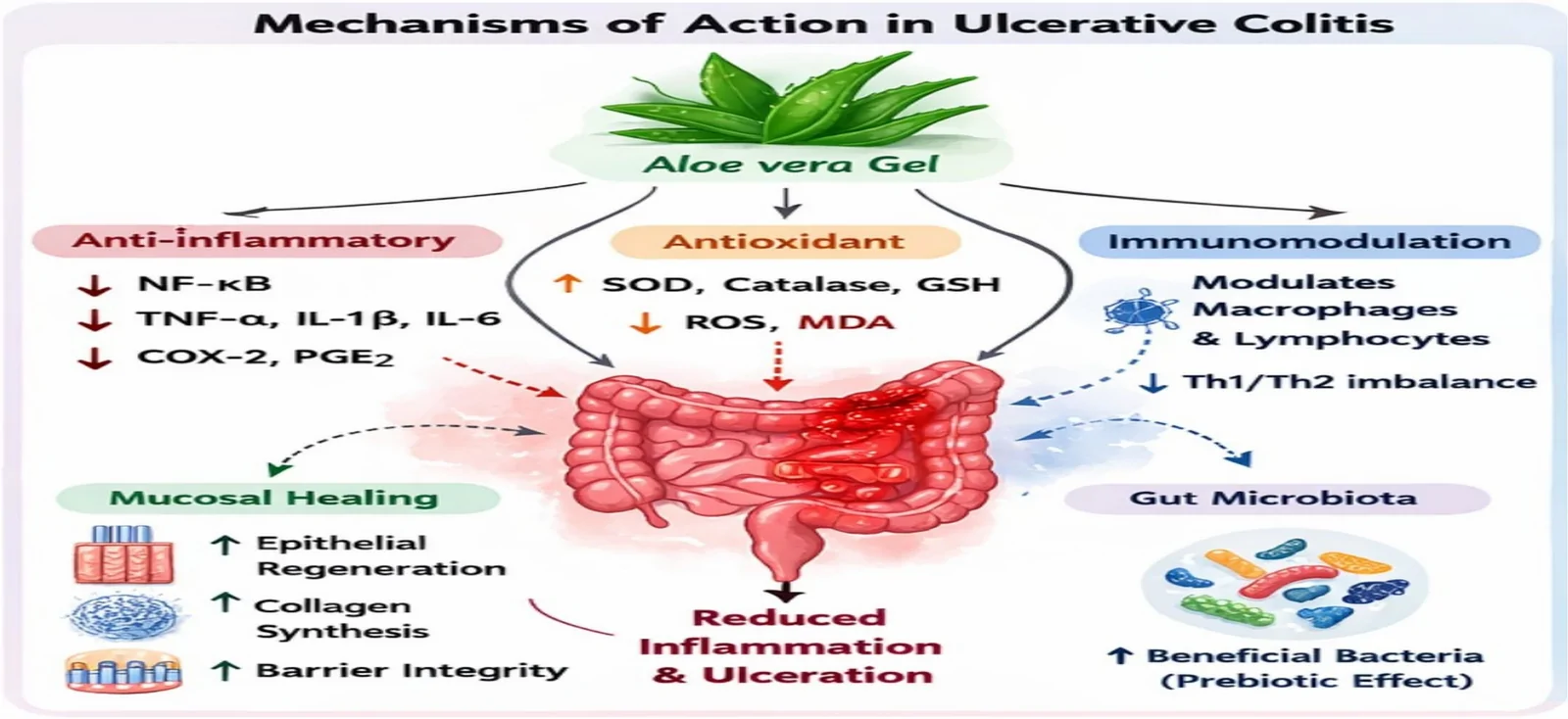
Mechanism of action of *Aleo vera* in UC (Babalola et al. 2022). Mechanisms of _Aloe vera_ gel in ulcerative colitis. _Aloe vera_ gel reduces colonic inflammation and ulceration via anti-inflammatory effects (↓NF-κB, TNF-α, IL-1β, IL-6, COX-2, PGE2), antioxidant activity (↑SOD, catalase, GSH; ↓ROS, MDA), and immunomodulation of macrophages, lymphocytes, and Th1/Th2 balance. It also promotes mucosal healing through epithelial regeneration, collagen synthesis, barrier integrity, and prebiotic effects on beneficial gut bacteria

Reduced expression of peroxisome proliferator-activated receptor gamma coactivator-1α (PGC-1α), a master regulator of mitochondrial biogenesis and energy metabolism, limits mitochondrial renewal and promotes the accumulation of dysfunctional mitochondria (Fig. 9). Consequently, immune cells undergo metabolic reprogramming, produce increased levels of inflammatory cytokines, and exacerbate tissue injury (Arya et al. 2025).

Emerging evidence further indicates that mitochondrial dysfunction alters gut microbial composition and impairs butyrate metabolism, thereby contributing to chronic intestinal inflammation and disease progression (Abu Shelbayeh et al. 2023).

#### Gut microbiota and colitis

The intestinal microbiota plays a fundamental role in maintaining immune homeostasis and epithelial barrier integrity. Beneficial microorganisms, including *Faecalibacterium prausnitzii, Bifidobacterium, Roseburia*, and *Lactobacillus* species, produce metabolites such as short-chain fatty acids (SCFAs), bile acid derivatives, and tryptophan metabolites that regulate immune function and preserve epithelial health.

In ulcerative colitis, depletion of these beneficial microorganisms together with expansion of pathogenic bacteria results in dysbiosis, impaired metabolite production, increased intestinal permeability, and exaggerated inflammatory responses. Restoration of microbial homeostasis has therefore emerged as an important therapeutic strategy for preventing disease progression and promoting mucosal healing (Alexeev et al. 2021; Liu et al. 2024).

### Clinical manifestations and diagnosis of colitis

The clinical presentation of ulcerative colitis (UC) varies according to disease severity and the extent of colonic involvement. The most common symptoms include abdominal pain or cramping, bloody diarrhea, rectal bleeding, fecal urgency, and tenesmus, which is characterized by the sensation of incomplete bowel evacuation. Patients with moderate-to-severe disease may also experience systemic manifestations such as fever, fatigue, malaise, anemia, and weight loss. In addition to intestinal symptoms, UC may be associated with extraintestinal manifestations involving the musculoskeletal, dermatological, ocular, and hepatobiliary systems, which can substantially affect patients' quality of life (Ungaro et al. 2017).

The diagnosis of UC is established through a combination of clinical evaluation, laboratory investigations, endoscopic examination, histopathological assessment, and imaging studies. Initial evaluation includes a detailed medical history and physical examination, followed by laboratory tests to exclude infectious causes of colitis. Measurement of inflammatory biomarkers, including C-reactive protein (CRP), erythrocyte sedimentation rate (ESR), and fecal calprotectin, provides valuable information regarding disease activity. Among these, fecal calprotectin is particularly useful for distinguishing inflammatory bowel disease from functional gastrointestinal disorders and for identifying patients who require endoscopic evaluation, especially in pediatric populations (Bakkaloğlu 2025).

Colonoscopy with mucosal biopsy remains the gold standard for diagnosing UC, allowing direct visualization of continuous mucosal inflammation and histopathological confirmation of the disease. Cross-sectional imaging modalities, including computed tomography (CT) and magnetic resonance imaging (MRI), are primarily reserved for evaluating disease extent, detecting complications, or excluding alternative diagnoses. In addition, emerging non-invasive diagnostic approaches, including fecal biomarkers, circulating microRNA profiling, and intestinal ultrasonography, have shown promise for improving early diagnosis, disease monitoring, and assessment of therapeutic response (Rashid et al. 2020).

## Current therapeutic strategies for ulcerative colitis

The management of colitis is determined by the underlying etiology, disease severity, and extent of intestinal involvement. In infectious colitis, treatment primarily consists of supportive care and targeted antimicrobial therapy when indicated. In contrast, the management of ulcerative colitis focuses on controlling intestinal inflammation, inducing and maintaining clinical remission, promoting mucosal healing, and preventing disease relapse. Current therapeutic approaches include conventional pharmacological agents, biologic therapies, small-molecule inhibitors, and, in selected cases, surgical intervention. Increasing interest has also been directed toward dietary modifications and plant-derived therapeutics as complementary strategies for disease management (Kaur and Goggolidou 2020).

### Conventional pharmacological therapies

Conventional pharmacological management of UC is guided by disease severity and therapeutic response.

Aminosalicylates (5-aminosalicylic acid; 5-ASA), including mesalazine (mesalamine) and sulfasalazine, are considered first-line therapy for patients with mild-to-moderate UC. These agents exert their anti-inflammatory effects by inhibiting cyclooxygenase (COX) and lipoxygenase (LOX) pathways, thereby reducing prostaglandin and leukotriene synthesis. Although generally well tolerated, adverse effects such as headache, nausea, abdominal discomfort, diarrhea, and skin rash may occur (Limsrivilai et al. 2025).

For patients with moderate-to-severe disease or acute exacerbations, corticosteroids, including prednisone and budesonide, are commonly used to induce remission. Their therapeutic effects are mediated through suppression of NF-κB signaling and inhibition of pro-inflammatory cytokine production. Despite their effectiveness, corticosteroids are unsuitable for long-term maintenance because of significant adverse effects, including osteoporosis, hyperglycemia, hypertension, cataracts, weight gain, acne, mood disturbances, increased appetite, and sleep disorders (Yamamoto-Furusho et al. 2017).

Immunomodulators, such as azathioprine, 6-mercaptopurine, and methotrexate, are primarily used as maintenance therapy in patients with steroid-dependent or frequently relapsing disease, particularly when treatment with 5-ASA is insufficient. These agents suppress lymphocyte proliferation and help maintain long-term remission; however, their delayed onset of action and potential adverse effects, including hepatotoxicity, nephrotoxicity, myelosuppression, and increased susceptibility to infections, require careful monitoring (Cai et al. 2021).

For patients with moderate-to-severe UC who fail to respond adequately to conventional therapy, biologic agents have significantly improved clinical outcomes. Anti-tumor necrosis factor-alpha (anti-TNF-α) monoclonal antibodies, including infliximab and adalimumab, inhibit TNF-α-mediated inflammatory signaling, whereas vedolizumab selectively blocks leukocyte trafficking through inhibition of α4β7 integrin, and ustekinumab targets the IL-12/IL-23 signaling pathway. In addition, small-molecule therapies, particularly Janus kinase (JAK) inhibitors, have emerged as effective oral treatment options for patients with refractory disease. Nevertheless, these therapies are associated with high costs and an increased risk of opportunistic infections and other immune-related adverse events (Limsrivilai et al., 2025; Vieujean et al. 2025).

The use of antimicrobial therapy is generally limited to infectious forms of colitis and is not routinely recommended for UC. Antibiotic selection depends on the causative pathogen and disease severity. For example, metronidazole may be used in selected bacterial infections, oral vancomycin is recommended for Clostridioides difficile infection, and valganciclovir is indicated for cytomegalovirus (CMV) colitis, with treatment duration guided by clinical response and laboratory findings (Al-Jashaami and Dupont 2016).

When medical therapy fails or life-threatening complications develop, surgical intervention, most commonly colectomy, may be required. Surgery is indicated in patients with refractory disease, toxic megacolon, perforation, severe hemorrhage, dysplasia, or colorectal cancer (Subramaniam et al. 2022).

Despite major advances in therapeutic management, UC remains a chronic relapsing–remitting disease associated with recurrent inflammation and progressive tissue injury. Serious complications include toxic megacolon, intestinal perforation, colorectal cancer, hemorrhage, strictures, and extraintestinal manifestations. Therefore, early diagnosis, individualized treatment, and long-term disease monitoring are essential for improving clinical outcomes and patients' quality of life (Holubar et al. 2021).

### Phytomedicine based natural products

The limitations associated with conventional therapies, including incomplete therapeutic response, adverse effects, high treatment costs, and the risk of relapse, have stimulated growing interest in plant-derived bioactive compounds as complementary or alternative approaches for the management of ulcerative colitis (UC). Medicinal plants contain diverse phytochemicals with antioxidant, anti-inflammatory, immunomodulatory, and microbiota-modulating properties that target multiple pathogenic mechanisms involved in UC. Unlike conventional drugs that often act on a single molecular target, phytochemicals frequently exert multi-target effects by simultaneously regulating inflammatory cytokines, oxidative stress, intestinal epithelial barrier integrity, and gut microbial homeostasis. Consequently, numerous medicinal plants have been investigated for their potential to attenuate intestinal inflammation and promote mucosal healing (Kciuk et al. 2024).

#### *Curcuma longa*

*Curcuma longa* L. (turmeric), a perennial herb belonging to the family Zingiberaceae, has been extensively used in traditional Ayurvedic and Chinese medicine for the treatment of inflammatory disorders. Its rhizomes are rich in biologically active constituents, including curcuminoids (curcumin, demethoxycurcumin, and bisdemethoxycurcumin), volatile oils (turmerones, atlantone, and zingiberene), polysaccharides, and phenolic compounds, which collectively contribute to its therapeutic properties (Gharge et al. 2021a).

Curcumin, the principal bioactive constituent, exhibits potent anti-inflammatory and antioxidant activities through modulation of multiple signaling pathways implicated in UC pathogenesis. Experimental studies have demonstrated that curcumin suppresses NF-κB activation, inhibits the expression of COX-2 and TNF-α (Gharge et al. 2021b), scavenges reactive oxygen species and enhances endogenous antioxidant enzymes including superoxide dismutase (SOD), catalase, and glutathione peroxidase (GPx). In addition, curcumin regulates immune responses (Sharifi-Rad et al. 2020).

In experimental models of dextran sulfate sodium and trinitrobenzene sulfonic acid-induced colitis, curcumin significantly reduced intestinal inflammation, oxidative stress, and histopathological injury. Its postulated mechanism of action is the suppression of NF-κB signaling pathway, along with favorable expression of Th1 and Th2 cytokines. Curcumin has also been reported to have anti-IL-1 and anti-TNF-α properties, which makes it an attractive naturopathic treatment option for inflammatory diseases such as UC. Furthermore, the concurrent use of curcumin with mesalamine yields a superior clinical and endoscopic response in the treatment of UC, strengthening the important role of natural components in UC treatment. (Chandan et al. 2020).

Clinical studies further suggest that curcumin supplementation, particularly when combined with mesalamine, improves both clinical remission and endoscopic outcomes in patients with UC. Besides curcuminoids, turmeric essential oils and polysaccharides may further enhance anti-inflammatory activity and intestinal barrier function, highlighting the therapeutic potential of *Curcuma longa* as an adjunctive treatment for inflammatory bowel disease (Opustilová et al. 2025).

#### *Boswellia serrata*

*Boswellia serrata* Roxb. (family Burseraceae) is another medicinal plant traditionally used for inflammatory and gastrointestinal disorders. Its pharmacological activity is primarily attributed to boswellic acids, particularly 11-keto-β-boswellic acid (KBA) and 3-O-acetyl-11-keto-β-boswellic acid (AKBA), together with other terpenoids present in the resin (Roy et al. 2019).

Boswellic acids exert anti-inflammatory effects mainly through selective inhibition of 5-lipoxygenase (5-LOX), thereby suppressing leukotriene biosynthesis and reducing inflammatory cell recruitment. They also inhibit NF-κB and MAPK signaling pathways, leading to decreased production of pro-inflammatory cytokines including TNF-α, IL-1β, and IL-6. Furthermore, Boswellia extracts attenuate oxidative stress by reducing lipid peroxidation, nitric oxide production, and inducible nitric oxide synthase (iNOS) expression while promoting epithelial barrier integrity and mucosal healing (Gupta et al. 2022).

**Fig. 10:**
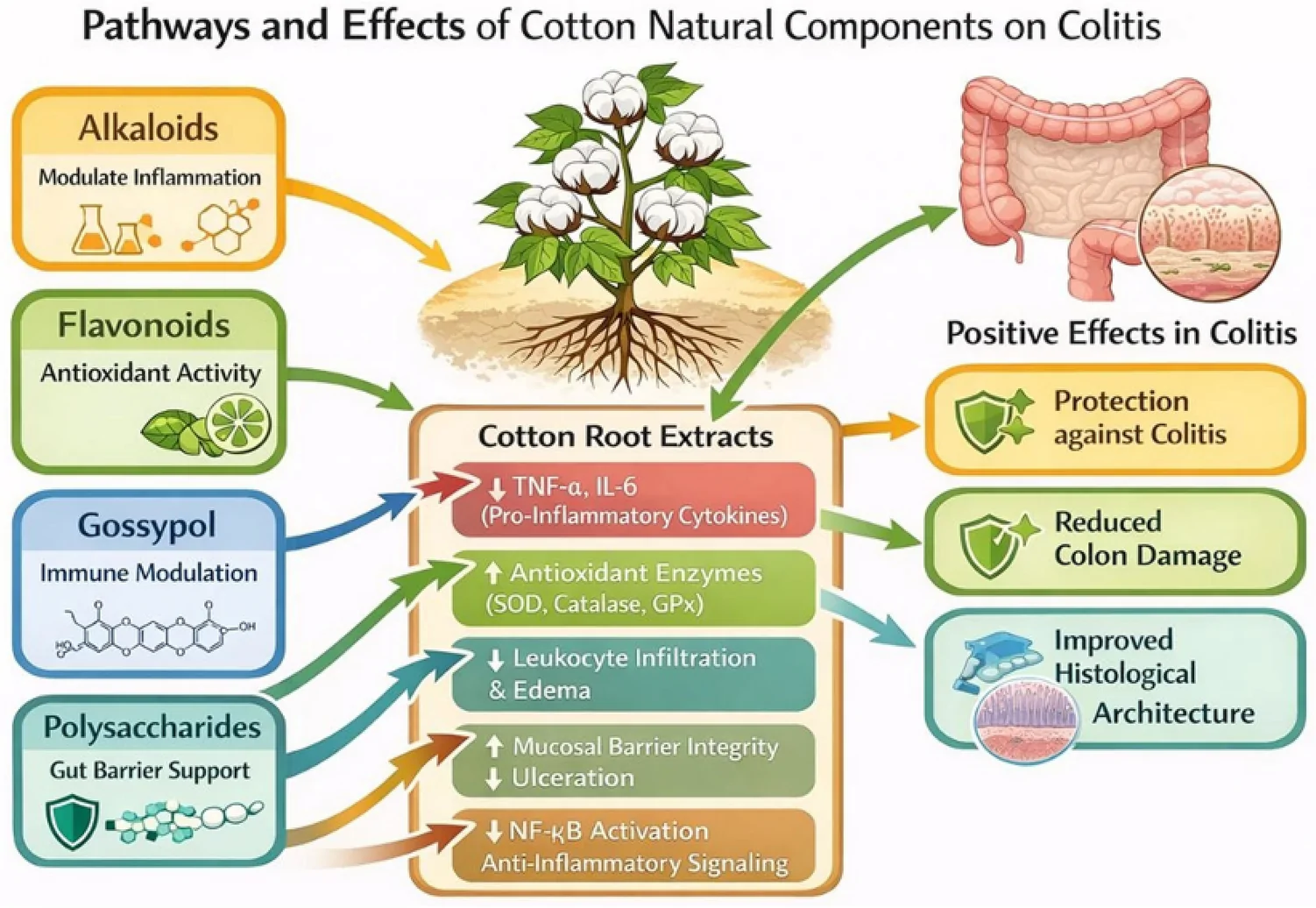
Pathways and effects of different classes of natural components of *G.B* on colitis (Olagoke et al. 2024). Cotton root extracts containing alkaloids, flavonoids, gossypol, and polysaccharides reduce colitis by suppressing TNF-α, IL-6, and NF-κB activation, enhancing antioxidant enzymes, and decreasing leukocyte infiltration and edema. These actions improve mucosal barrier integrity, reduce ulceration and colon damage, and restore histological architecture

Preclinical studies consistently demonstrate that *Boswellia serrata* extract reduces macroscopic and microscopic damage, preserves crypt architecture, and alleviates tissue injury in experimental colitis. Computational investigations and analyses further predict strong interactions with key inflammatory targets, including COX-1, COX-2, and iNOS, supporting its multi-target pharmacological profile (Fig. 10). Additionally, *Boswellia serrata* constituents demonstrate good ADME features and a favorable toxicity profile, executing gastroprotective and intestinal anti-inflammatory actions through antioxidant and mucosal protective mechanisms (Serafim et al. 2026). Although clinical studies suggest favorable efficacy and safety, variations in extract composition and bioavailability remain major challenges. Therefore, standardized formulations and large-scale randomized clinical trials are required before routine clinical application can be recommended (Roy et al. 2019 and Governa et al. 2018).

#### Aloe vera

*Aloe vera* is a medicinal plant recognized for its anti-inflammatory, antioxidant, immunomodulatory, and wound-healing activities. Its therapeutic effects are primarily attributed to the inner gel, which contains polysaccharides such as acemannan, together with glucomannans, phenolic compounds, vitamins, sterols, and antioxidant enzymes (Naini et al. 2021).

Experimental evidence indicates that *Aloe vera* gel attenuates intestinal inflammation by suppressing NF-κB signaling, reducing the production of TNF-α, IL-1β, and IL-6, and enhancing endogenous antioxidant defenses. Acemannan additionally promotes macrophage regulation, epithelial regeneration, collagen synthesis, and restoration of intestinal barrier integrity, thereby facilitating mucosal healing (Sánchez et al. 2020).

In animal models of chemically induced colitis, particularly acetic acid and DSS-induced colitis models, treatment with gel derived from *Aloe vera* has demonstrated significant therapeutic effects. Administration of *Aloe vera* gel was associated with a reduction in the disease activity index and attenuation of colon shortening. Additionally, histological examination revealed improved mucosal architecture and reduced inflammatory cell infiltration, indicating enhanced tissue repair and protection against colonic damage. These findings collectively suggest that *Aloe vera* exerts anti-inflammatory and mucosal healing effects, supporting its potential as a complementary therapeutic agent for UC in experimental settings (Bahrami et al. 2020).

Although *Aloe vera* gel is generally considered safe, prolonged exposure to anthraquinone-rich latex preparations may cause gastrointestinal irritation and electrolyte imbalance. Consequently, standardized gel preparations are preferred for therapeutic use, while additional well-designed clinical trials are needed to establish optimal dosage, long-term safety, and clinical efficacy in patients with UC (Sánchez et al. 2020).

#### *Gossypium barbadense* L.: a promising phytotherapeutic candidate for ulcerative colitis

*Gossypium* is a genus of flowering plants belonging to the family Malvaceae, comprising more than 50 species distributed throughout tropical and subtropical regions of Africa, Asia, Australia, and the Americas. Besides its global economic importance as the principal source of natural cotton fiber, the genus has attracted considerable scientific interest because of its rich repertoire of secondary metabolites with diverse biological activities (Anwar et al. 2022b).

Among these species, *Gossypium barbadense* L. (Egyptian cotton) has emerged as a promising medicinal plant owing to its abundance of bioactive phytochemicals, including flavonoids, polyphenols, terpenoids, alkaloids, tannins, saponins, and glycosides. These constituents exhibit potent antioxidant, anti-inflammatory, antimicrobial, and immunomodulatory properties, suggesting potential therapeutic applications in chronic inflammatory diseases, including ulcerative colitis (Mohammed et al. 2024).

Although phytochemicals are distributed throughout the plant, the root represents one of the richest sources of defensive secondary metabolites. Phytochemical analyses have identified numerous bioactive compounds in *G. barbadense* roots, including sesquiterpene aldehydes, flavonoids, phenolic acids, tannins, alkaloids, saponins, and glycosides (Egbuta et al. 2017). Collectively, these compounds act on multiple pathological mechanisms implicated in UC by suppressing inflammatory signaling, reducing oxidative stress, preserving intestinal epithelial barrier integrity, and modulating immune responses.

The phytochemical composition of G. barbadense root is influenced by cultivar, environmental conditions, plant maturity, and extraction procedures. Consequently, comprehensive phytochemical characterization using advanced analytical techniques such as HPLC, GC–MS, and LC–MS/MS is essential for standardizing extracts, identifying active constituents, and facilitating pharmacological and toxicological investigations (Pal et al. 2022).

##### Major bioactive phytochemicals and their therapeutic relevance

The major phytochemical classes identified in *G. barbadense* root contribute to its therapeutic efficacy by directly targeting specific pathogenic mechanisms in ulcerative colitis.

###### Terpenoids

Terpenoids constitute one of the most pharmacologically important groups of compounds present in G. barbadense roots. Among them, gossypol, a sesquiterpene polyphenolic aldehyde, has received particular attention because of its broad spectrum of biological activities. At carefully controlled concentrations, gossypol exhibits antioxidant, antimicrobial, anti-inflammatory, and immunomodulatory properties, although its dose-dependent toxicity necessitates careful evaluation before clinical application (Pal et al. 2022).

Experimental studies suggest that gossypol attenuates intestinal inflammation by suppressing NF-κB activation, thereby reducing the production of pro-inflammatory cytokines including TNF-α, IL-1β, and IL-6. In addition, it downregulates COX-2 and inducible nitric oxide synthase (iNOS), decreases nitric oxide and prostaglandin production, and limits macrophage and neutrophil activation, ultimately reducing mucosal inflammation (Saleh et al. 2021 and Shi et al. 2024).

Beyond its anti-inflammatory effects, gossypol exhibits antioxidant activity through scavenging reactive oxygen species and limiting oxidative tissue injury, thereby preserving epithelial architecture. As a BH3 mimetic, it may also promote apoptosis of activated inflammatory cells, contributing to the restoration of immune homeostasis. Furthermore, inhibition of COX-2 and LOX pathways reduces prostaglandin and leukotriene synthesis, providing additional anti-inflammatory activity comparable to several conventional therapeutic strategies used in UC (Zheng et al. 2025). Other terpenoids, including β-caryophyllene, limonene, α-pinene, and farnesol, may further contribute to the overall anti-inflammatory efficacy of G. barbadense root extracts (Chen & Boskovic 2024).

###### Phenolic compounds

Phenolic acids, including caffeic, ferulic, and p-coumaric acids, represent another important class of phytochemicals in *G. barbadense* roots. These compounds exert potent antioxidant effects through free-radical scavenging, metal chelation, and inhibition of lipid peroxidation. By limiting oxidative stress and modulating inflammatory signaling pathways, phenolic compounds may protect colonic tissues against inflammation-induced injury and contribute to preservation of intestinal barrier function (Nurzyńska-Wierdak 2023 and El-mousalamy et al. 2023).

###### Flavonoids

Flavonoids, particularly the cotton-specific flavonols gossypetin and gossypin, are abundant constituents of *G. barbadense* root extracts. These compounds possess well-established antioxidant and anti-inflammatory properties and have been shown to suppress reactive oxygen species, inhibit pro-inflammatory cytokine production, and regulate NF-κB and MAPK signaling pathways. Through these mechanisms, flavonoids reduce epithelial injury, preserve tight junction integrity, and promote mucosal healing in experimental models of colitis (Panche et al. 2016).

###### Alkaloids

Alkaloids represent another important class of nitrogen-containing phytochemicals identified in *Gossypium barbadense* root extracts. These compounds have been reported to regulate inflammatory signaling pathways by suppressing pro-inflammatory cytokine production and attenuating oxidative stress (Kang and Kim 2023). Among the alkaloid-like constituents, choline has attracted particular interest because of its physiological and pharmacological functions. As a precursor of phosphatidylcholine, choline is essential for maintaining cellular membrane integrity, especially within the intestinal epithelium. Moreover, activation of the cholinergic anti-inflammatory pathway by choline suppresses the production of inflammatory cytokines, including TNF-α and IL-6, while enhancing epithelial barrier function and protecting intestinal tissues against oxidative and inflammatory injury. These combined effects suggest that alkaloid constituents may contribute to the protective activity of G. barbadense root in inflammatory disorders such as ulcerative colitis (Burns et al. 2025 and Muhammad et al. 2014).

###### Tannins

Tannins constitute another major group of polyphenolic compounds present in *Gossypium barbadense*, predominantly as condensed tannins (proanthocyanidins), with smaller amounts of hydrolysable tannins reported in root and bark tissues (Ade-Ademilua and Okpoma 2018 and El-mousalamy et al. 2023). These compounds possess strong antioxidant and anti-inflammatory activities through free-radical scavenging, inhibition of lipid peroxidation, and stabilization of cellular membranes. Experimental studies indicate that proanthocyanidins attenuate intestinal inflammation by reducing oxidative stress, suppressing inflammatory mediators such as TNF-α, and strengthening the intestinal epithelial barrier, thereby limiting mucosal injury associated with ulcerative colitis (Cosme et al. 2025 and Egbuta et al. 2017).

###### Saponins and glycosides

Saponins and glycosides further contribute to the pharmacological profile of G. barbadense root through complementary antioxidant, anti-inflammatory, immunomodulatory, and gastroprotective activities. Saponins have been reported to stabilize cellular membranes, suppress the production of pro-inflammatory cytokines (TNF-α, IL-1β, and IL-6), enhance endogenous antioxidant defenses, and regulate immune-cell activation within the intestinal mucosa. Although these compounds may exhibit mild hemolytic activity at high concentrations, they are considered important contributors to the anti-inflammatory effects of botanical extracts (Tang et al. 2025; Mieres-Castro and Mora-Poblete 2023).

Flavonoid glycosides, including quercetin and kaempferol glycosides, exert potent cytoprotective effects by scavenging reactive oxygen species, inhibiting NF-κB and MAPK signaling pathways, preserving tight junction proteins, and promoting epithelial regeneration. In addition, these compounds enhance mucus secretion and favorably modulate gut microbiota, thereby supporting restoration of intestinal homeostasis and mucosal healing **(**Vezza et al. 2016).

##### Integrated therapeutic potential of *Gossypium barbadense* root

Collectively, the diverse phytochemical constituents of *Gossypium barbadense* root (including terpenoids, phenolic compounds, flavonoids, alkaloids, tannins, saponins, and glycosides) appear to act synergistically to target multiple pathogenic mechanisms involved in ulcerative colitis. Experimental evidence indicates that these phytochemicals reduce oxidative stress by lowering malondialdehyde (MDA) levels while enhancing endogenous antioxidant enzymes such as superoxide dismutase (SOD), catalase, and glutathione peroxidase (GPx) (Jomova et al. 2024). They also improve colonic histopathology by reducing epithelial damage, preserving mucosal architecture, promoting intestinal barrier integrity, and restoring microbial homeostasis (Guo and Mei 2016).

Comprehensive phytochemical analyses using LC–MS/MS and GC–MS have identified more than 50 bioactive constituents in *G. barbadense* root extracts, including gossypetin, gossypolone derivatives, coumarins, eugenol derivatives, and palmitic and linoleic acid derivatives, many of which possess documented antioxidant and anti-inflammatory activities (Teoh et al. 2023). Importantly, several constituents have been associated with increased expression of the tight junction proteins occludin and claudin, which are essential for maintaining intestinal epithelial barrier integrity and limiting bacterial translocation during intestinal inflammation (Li et al. 2022). Furthermore, modulation of Th1/Th2 immune responses suggests an additional immunoregulatory mechanism that may contribute to sustained resolution of chronic intestinal inflammation (Alamgir 2018).

Overall, the therapeutic potential of *G. barbadense* root is likely attributable to the synergistic interactions among its multiple phytochemical constituents rather than the activity of a single compound. This multi-component, multi-target mode of action distinguishes *G. barbadense* from many conventional single-target therapies and provides a strong rationale for further pharmacological investigation, network pharmacology analysis, and clinical evaluation as a complementary therapeutic strategy for ulcerative colitis (Sun et al. 2025).

##### Toxicological profile and safety considerations

Despite its therapeutic potential, the clinical translation of *Gossypium barbadense* requires careful evaluation of its native toxic constituent, gossypol. Free gossypol acts as a natural plant defense mechanism but poses risks of systemic toxicity, including anorexia, respiratory distress, weight impairment, cellular damage, reversible or irreversible male infertility, and hypokalemia. Clinical observations have also noted side effects such as hemolytic anemia, diarrhea, and gastrointestinal distress. To mitigate these risks, several physical, chemical, and microbial detoxification methods—including thermal processing, alkali immersion, and fermentation can be deployed to reduce free gossypol content. Furthermore, managing toxicities can be achieved clinically through precise dose reduction and symptomatic treatment, while chemical derivatization of the gossypol scaffold offers a viable structural strategy to enhance biological efficacy and minimizing inherent toxicity **(**Paunovic et al. 2023).

## Network pharmacology-based investigation

### Molecular mechanisms underlying the anti-colitic activity of *Gossypium barbadense*

Network pharmacology has emerged as an important systems biology strategy for elucidating the complex interactions between bioactive compounds, molecular targets, signaling pathways, and disease phenotypes. Unlike the traditional “one drug–one target” paradigm, network pharmacology recognizes that herbal medicines contain multiple bioactive constituents capable of simultaneously regulating numerous molecular targets and interconnected biological pathways. This multi-component, multi-target mode of action is particularly suitable for complex inflammatory disorders such as ulcerative colitis (UC), in which multiple signaling cascades including inflammatory cytokines, oxidative stress, epithelial barrier dysfunction, immune dysregulation, and programmed cell death that contribute collectively to disease progression (Zhai et al. 2025).

Recent advances in computational biology have enabled the integration of phytochemical profiling with target prediction, protein interaction networks, functional enrichment analyses and pathway mapping. Public databases such as PubChem, ChEMBL, SwissTargetPrediction, GeneCards, ChemSpider, KNApSAcK, BATMAN-TCM, STRING, KEGG, and Gene Ontology (GO) provide valuable resources for identifying compound–target interactions and predicting the molecular mechanisms underlying botanical medicines (Muthuramalingam et al. 2024). Integration of these resources with network visualization software, particularly Cytoscape, enables identification of hub genes, critical signaling pathways, and biological processes that may represent key therapeutic targets.

The application of network pharmacology has become increasingly valuable for medicinal plant research because it provides mechanistic evidence supporting the traditional therapeutic use of herbal extracts while facilitating the discovery of novel drug candidates. When combined with phytochemical characterization techniques such as LC–MS/MS or GC–MS, this approach allows comprehensive mapping of phytoconstituents to disease-associated molecular networks, thereby accelerating the identification of pharmacologically active compounds and their potential mechanisms of action.

Among the phytochemicals identified in *Gossypium barbadense* root, several compounds have previously demonstrated anti-inflammatory activities through mechanisms consistent with UC therapy. For example, syringic acid, identified by LC–MS analysis in cotton root extracts, has been reported to attenuate experimental colitis by suppressing activation of the NLRP3/Caspase-1/GSDMD/IL-1β inflammasome pathway while simultaneously modulating gut microbiota composition. These findings illustrate how individual phytochemicals contribute to the broader poly-pharmacological profile of *G. barbadense* and support the application of network pharmacology for mechanistic investigation (Abiola et al. 2025).

### Workflow of the network pharmacology analysis

**Fig. 11:**
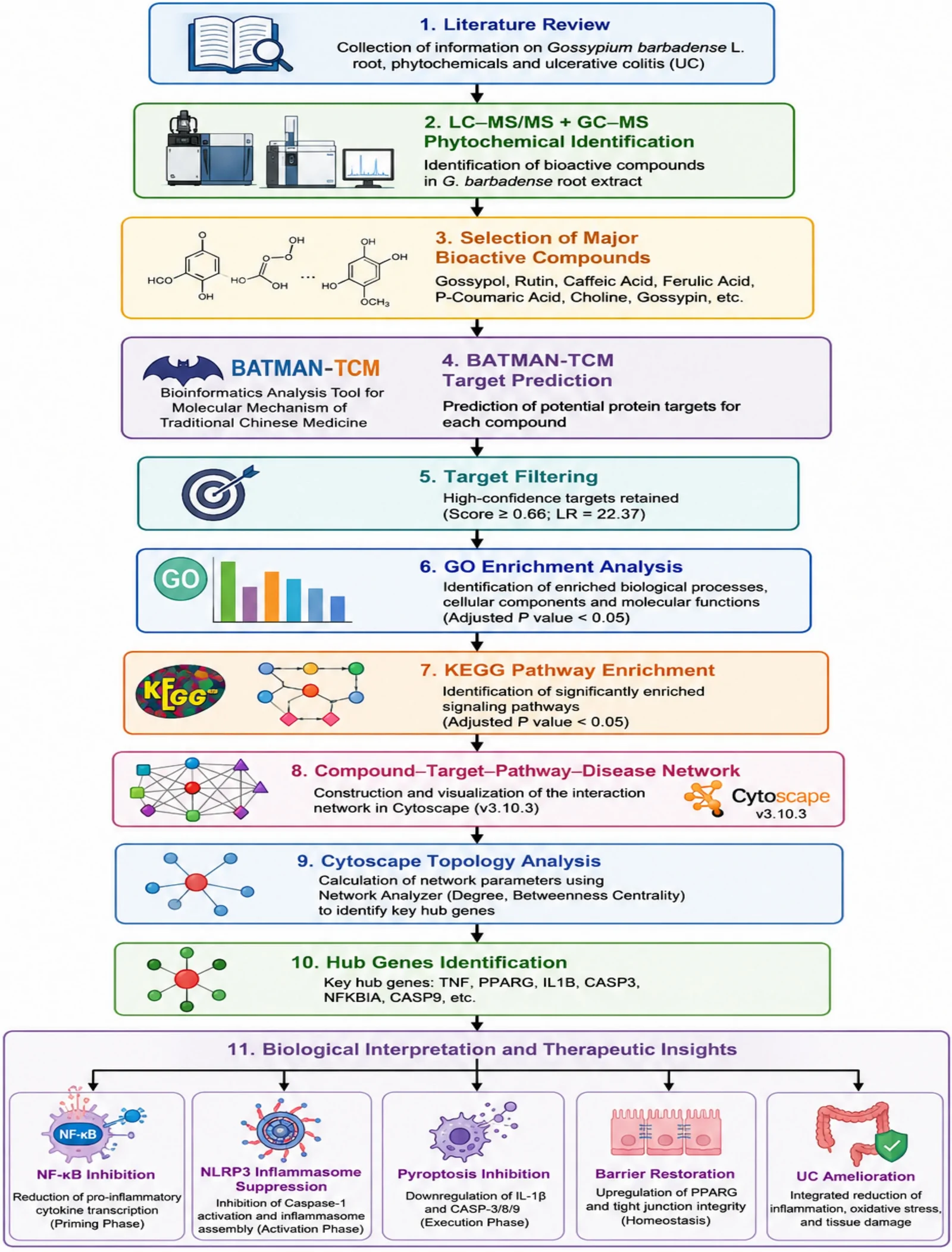
Integrated network pharmacology workflow for investigating the molecular mechanisms of *Gossypium barbadense* L. root extract in ulcerative colitis. The workflow illustrates the sequential computational strategy, beginning with the identification of bioactive phytochemicals from *G. barbadense* root, followed by compound structure retrieval and target prediction using BATMAN-TCM. Functional enrichment analyses (GO and KEGG) were then performed to identify significantly associated biological processes and signaling pathways. Finally, Cytoscape was used to construct and analyze the compound–target–pathway–disease network, identifying TNF, IL1B, PPARG, NFKBIA, and CASP3 as major hub genes involved in the regulation of NF-κB signaling, NLRP3 inflammasome activation, apoptosis/pyroptosis, oxidative stress, and mucosal barrier repair, thereby providing a systems-level explanation for the therapeutic effects of *G. barbadense* in ulcerative colitis

To elucidate the molecular basis underlying the therapeutic potential of *Gossypium barbadense* root against UC, a comprehensive network pharmacology workflow was performed (Fig.11). Major phytochemicals identified from the literature and phytochemical profiling (such as gossypol, rutin, caffeic acid, ferulic acid, p-coumaric acid, choline, and gossypin) were analyzed using the BATMAN-TCM (Bioinformatics Analysis Tool for Molecular Mechanism of Traditional Chinese Medicine) platform. Potential protein targets were predicted using a stringent likelihood score threshold (score ≥ 0.66; LR = 22.37), thereby retaining only high-confidence interactions.

Subsequently, Gene Ontology (GO) enrichment analysis and Kyoto Encyclopedia of Genes and Genomes (KEGG) pathway enrichment were conducted to identify the principal biological processes and signaling pathways regulated by the predicted targets. Statistical significance was defined as an adjusted *P-value* < 0.05. The resulting compound–target–pathway–disease interaction network was visualized using Cytoscape (v 3.10.3), while network topology parameters, including degree centrality and betweenness centrality, were calculated using the Network Analyzer plugin to identify key regulatory hub genes involved in UC pathogenesis.

This integrative computational strategy provides a systems-level understanding of how multiple phytochemicals within *Gossypium barbadense* root may cooperatively regulate inflammatory signaling, oxidative stress, epithelial barrier integrity, apoptosis, and inflammasome activation. Such an approach complements conventional phytochemical studies by providing mechanistic evidence supporting the therapeutic application of *G. barbadense* as a promising network-level therapeutic intervention for ulcerative colitis.

### Identification of bioactive compounds and molecular targets

The molecular mechanisms underlying the therapeutic potential of *Gossypium barbadense* root, a network pharmacology approach was employed using the BATMAN-TCM platform. Seven major phytochemicals, including gossypin, gossypol, rutin, ferulic acid, caffeic acid, *p*-coumaric acid, and choline, were screened to identify their experimentally validated and computationally predicted protein targets.

A total of 213 unique molecular targets were identified (Table 1), indicating that *G. barbadense* exhibits a typical multi-component, multi-target pharmacological profile. Several compounds demonstrated broad target specificity. Rutin possessed the largest number of experimentally validated targets (n = 33), whereas caffeic acid and choline interacted with 25 and 16 known targets, respectively. Gossypol exhibited extensive interactions with apoptosis-related proteins, including CASP3, CASP8, CASP9, and BCL2, together with inflammatory mediators such as ICAM1 and CXCL8. Likewise, caffeic acid and *p*-coumaric acid targeted key inflammatory regulators including TNF, TLR4, PTGS2, NFKB1, and MAPK1, supporting their potential anti-inflammatory activities.

**Table 1:** Bioactive compounds of *G. barbadense* and their associated molecular targets

Compound	No. of known target proteins	Known target proteins	No. of predicted target proteins	Predicted target proteins (descending order by score)
RUTIN	33	CASP3, S100A8, MAPK14, P4HB, PPARG, PPARGC1A, TFAM, FN1, BCL2, HMGCR, STAT4, FAS, CAT, CBR1, UCP1, MAPK8, MTOR, MPO, IL1B, SIRT1, IFNG, INS, CXCL1, BACE1, ADA, MARK4, ACTA2, TNF, TGFB1, NR1H4, AKR1C3, IL6, PROCR	17	AKR1C2^0.99^, AKR1C4^0.99^, AKR1C1^0.99^, AKR1D1^0.95^, RDH10^0.78^, HSD17B6^0.78^, HSD17B1^0.78^, PTGFR^0.78^, PTGDR^0.78^, HSD3B1^0.78^, SRD5A1^0.78^, PDE2A^0.78^, SRD5A3^0.78^, AKR1A1^0.74^, CYP1B1^0.7^, ESRRB^0.7^, ESRRA^0.7^
Caffeic Acid	25	CYP2C9, SELP, RAC1, HMOX1, ALOX5, CYP1A1, BCHE, CTSC, KCNN4, NFKB1, PTGS1, TLR4, TOP1, TNF, CYP2D6, CYP3A4, MAPK3, GFAP, PTGS2, PRKCB, CYP1A2, CYP2C19, MAPK1, ACHE, NFE2L2	6	ARF1^0.72^, PADI4^0.72^, PNLIP^0.72^, DDC^0.72^, AR^0.72^, IFNG^0.68^
Choline	16	PHOSPHO1, MAPK14, BCHE, CHRFAM7A, SIGMAR1, CHRNA4, PLD1, ACHE, PCYT1B, HMOX1, CHRNA7, PEMT, PLD2, TNF, VEGFC, PCYT1A	20	CHRM3^0.92^, CHRM4^0.92^, CHRM1^0.92^, CHRM2^0.92^, ADH1B^0.88^, RHO^0.88^, GSTZ1^0.88^, GABBR1^0.88^, ADH1A^0.88^, ADH1C^0.88^, GATM^0.88^, NAGA^0.88^, PPT1^0.88^, FOLR1^0.88^, ARF1^0.88^, HNF4G^0.88^, GABBR2^0.88^, PPARA^0.88^, PPARD^0.88^, ALPL^0.82^
Ferulic Acid	16	CYP2C9, ENO2, PRKAA1, MAP2K1, APP, PVALB, CTNNB1, BCHE, PEA15, MAP2K2, PRDX2, HPCA, CXCL8, MAOA, ACHE, NFE2L2	3	GSTP1^0.8^, PPARG^0.8^, VDR^0.8^
Gossypin	15	MMP9, LDLR, XIAP, PTP4A3, BCL2L1, BIRC2, CCND1, MYC, BCL2, RELA, VEGFA, PTGS2, BIRC5, NFKBIA, MAPK1	6	AKR1C1^0.68^, AKR1C2^0.68^, AR^0.68^, RDH10^0.66^, AKR1D1^0.66^, SRD5A3^0.66^
Gossypol	14	CASP3, CASP9, CXCL8, BCL2, NCOA3, BCL2L1, CASP8, GJA1, NCOA1, ICAM1, HSD11B1, PLG, APEX1, LDHA	20	HDAC6^0.88^, HDAC8^0.88^, HDAC1^0.88^, HDAC2^0.88^, HDAC10^0.88^, HDAC3^0.88^, HDAC11^0.88^, HDAC5^0.88^, HDAC4^0.88^, HDAC9^0.88^, HTR1D^0.72^, CACNA1C^0.72^, HTR6^0.72^, CACNA1S^0.72^, OPRD1^0.72^, ADRA2B^0.72^, ADRA1A^0.72^, PPARG^0.72^, SMPD1^0.72^, SLC6A3^0.72^
P-Coumaric Acid	7	MMP9, CCL16, BACE1, TYR, PTPN1, MYC, NOS3	15	IFNG^0.84^, TNF^0.82^, AKR1C1^0.68^, CHUK^0.68^, ALOX5^0.68^, AKR1C2^0.68^, SLC7A11^0.68^, PTGS1^0.68^, PPARG^0.68^, PTGS2^0.68^, ACAT1^0.68^, NOS2^0.68^, CCL2^0.66^, IL1B^0.66^, SYK^0.66^

These findings demonstrate that the major phytochemicals present in *G. barbadense* root collectively target multiple biological processes involved in ulcerative colitis rather than acting through a single molecular pathway.

### Protein interaction network reveals TNF as the principal hub gene

**Fig. 12:**
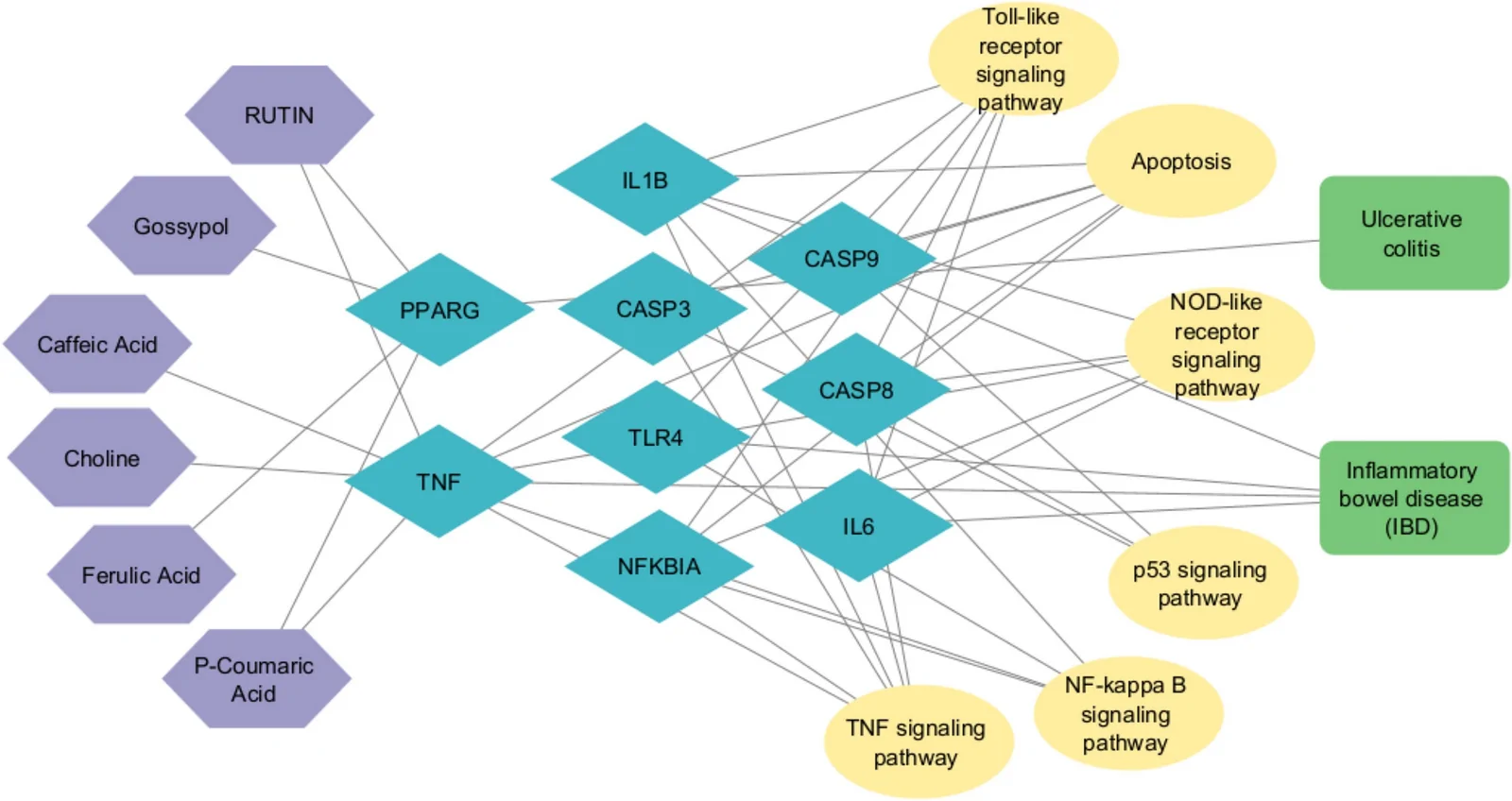
Compound-Target-Pathway-Disease Interactome. The network represents the multi-target mechanisms of G. barbadense in the treatment of UC. Purple hexagons represent the primary bioactive ingredients (e.g., Gossypol, Rutin, Caffeic Acid, and Choline). Teal diamonds represent predicted protein targets, with the node size corresponding to the degree centrality; larger nodes such as TNF (Degree: 10) and PPARG (Degree: 5) indicate central hub genes within the network. Yellow/Tan ellipses denote significantly enriched signaling pathways (Padj < 0.05), highlighting the NOD-like receptor, NF-kB, and apoptosis cascades. Green rectangles represent the clinical endpoints, specifically UC and IBD. Edges indicate documented or high-confidence predicted interactions between nodes

Protein–protein interaction (PPI) network analysis identified several highly connected hub genes that may play central roles in mediating the biological activity of *G. barbadense* (Fig. 12; Table 2).

**Table 2:** Topological parameters of key hub genes

Rank	Hub Gene (Symbol)	Degree (Total edges)	Betweenness Centrality	Biological Role in UC
1	TNF	10	0.610	Master Inflammatory Regulator
2	IL1B	6	0.000	Pyroptosis Execution Cytokine
3	PPARG	5	0.110	Anti-inflammatory Transcription Factor
4	NFKBIA	5	0.000	NF-kappaB Signaling Inhibitor
5	CASP3	3	0.000	Executioner of Apoptosis/Pyroptosis

Among all identified targets, TNF emerged as the dominant hub, exhibiting the highest degree (10) and betweenness centrality (0.610), indicating its central position within the inflammatory interaction network. Other prominent hub genes included IL1B (degree = 6), PPARG (degree = 5), NFKBIA (degree = 5), and CASP3 (degree = 3). These proteins are recognized regulators of inflammatory signaling, epithelial homeostasis, and programmed cell death, suggesting that they represent major molecular targets of *G. barbadense* bioactive compounds.

The prominent network connectivity of TNF and PPARG further indicates that these proteins may function as key regulatory nodes through which multiple phytochemicals exert coordinated therapeutic effects.

### Functional enrichment analysis

Functional enrichment analysis demonstrated that the identified targets were significantly associated with biological processes involved in inflammation, cell proliferation, and programmed cell death.

Among the enriched KEGG pathways (Table 3), the NFKB signaling pathway (adjusted *P* = 1.64 × 10⁻^3^) showed the strongest association with inflammatory regulation, whereas the NOD-like receptor signaling pathway (adjusted *P* = 4.27 × 10⁻^2^) was significantly enriched among genes involved in inflammasome activation and pyroptosis. Additional enrichment was observed for the HIF-1 signaling pathway, suggesting a potential role in regulating oxidative stress and intestinal hypoxia. Steroid hormone biosynthesis was also significantly represented, indicating possible involvement in epithelial repair and restoration of mucosal integrity.

**Table 3:** Bioinformatic Enrichment analysis of *G. barbadense* targets

Analysis Category	Pathway / Disease Name	Adjusted* P*-value	Biological Relevance
Disease Match	Inflammatory Bowel Disease	4.98×10^−2^	Direct clinical validation for colitis
Cell Death	NOD-like Receptor Signaling	4.27×10^−2^	Confirms the mechanism of pyroptosis
Primary Hub	NF-kappa B Signaling Pathway	1.64×10^−3^	Controls the “priming” of inflammation
Tissue Repair	Steroid Hormone Biosynthesis	5.71×10^−11^	Potential for mucosal barrier restoration
Stress Response	HIF-1 Signaling Pathway	5.26×10^−5^	Protection against gut hypoxia/oxidative stress

Disease enrichment analysis identified inflammatory bowel disease as the most relevant disease category, supporting the biological relevance of the predicted molecular targets.

### Mapping of molecular targets onto the NOD-like receptor signaling pathway

**Fig. 13:**
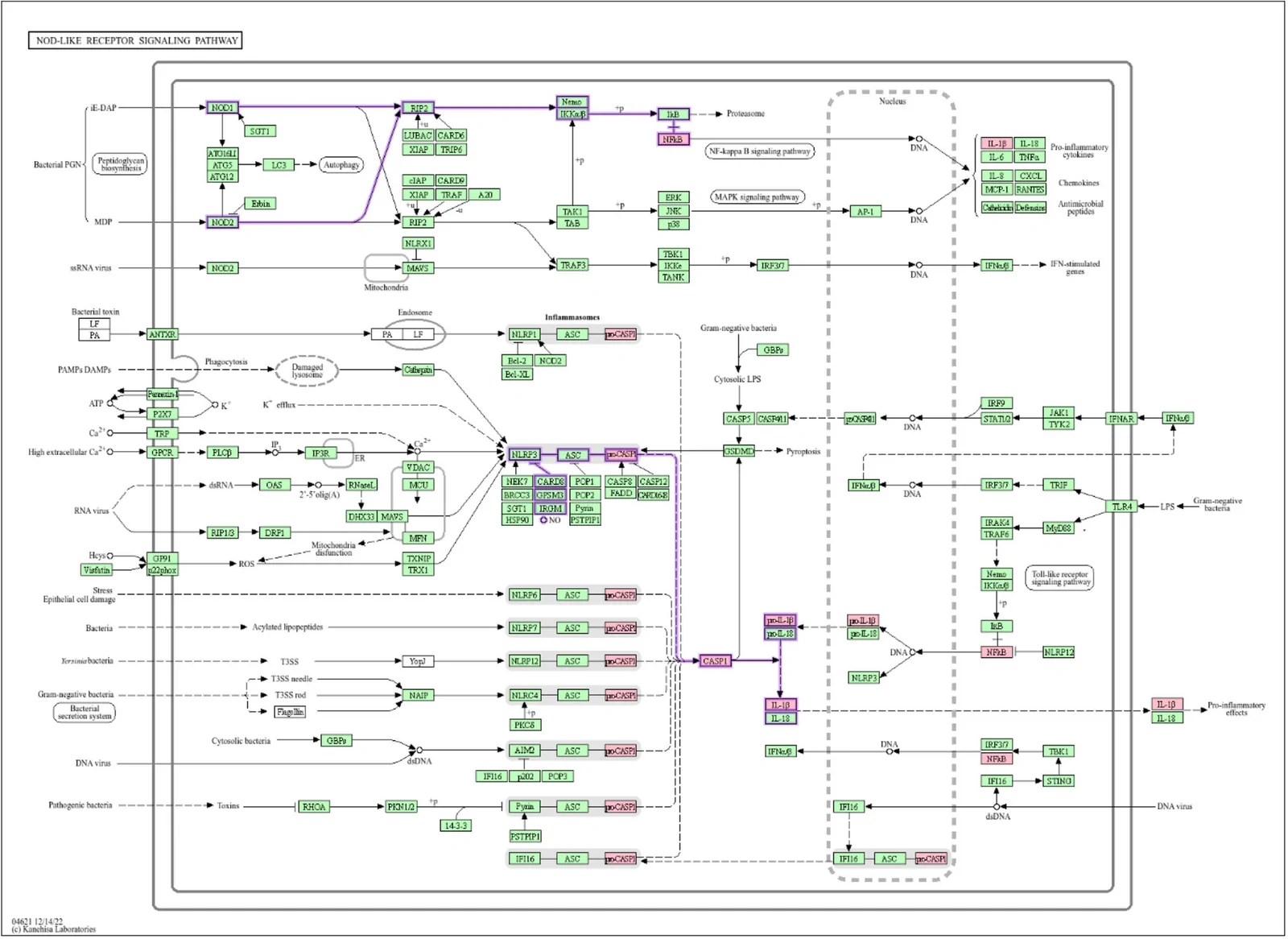
Mechanistic Mapping of *G. barbadense* Targets on the NOD-like Receptor Signaling Pathway Pink Highlighted Boxes: Indicate the core therapeutic targets of the root extract identified through the network pharmacology pipeline. NF-kappaB Signaling: The highlighting of the NF-kappaB complex confirms the extract's role in the “priming” phase of inflammation, regulating the transcription of pro-inflammatory precursors. Pyroptosis Execution: The simultaneous highlighting of CASP-1 (Caspase-1) and IL-1B (Interleukin-1beta) maps the extract's intervention to the formation of the inflammasome and the subsequent execution of pyroptosis. Clinical Implications: This mapping demonstrates that *G. barbadense* does not just reduce general inflammation but specifically interrupts the programmed cell death pathway responsible for epithelial barrier breakdown in ulcerative colitis

To further investigate the mechanisms associated with inflammasome regulation, the predicted targets were mapped onto the KEGG NOD-like receptor signaling pathway (hsa04621) (Fig. 13).

The analysis demonstrated a marked concentration of target genes around the NF-κB, CASP1, and IL1B signaling nodes. These molecules represent critical regulatory checkpoints involved in both the priming and activation phases of inflammasome signaling.

**Fig. 14:**
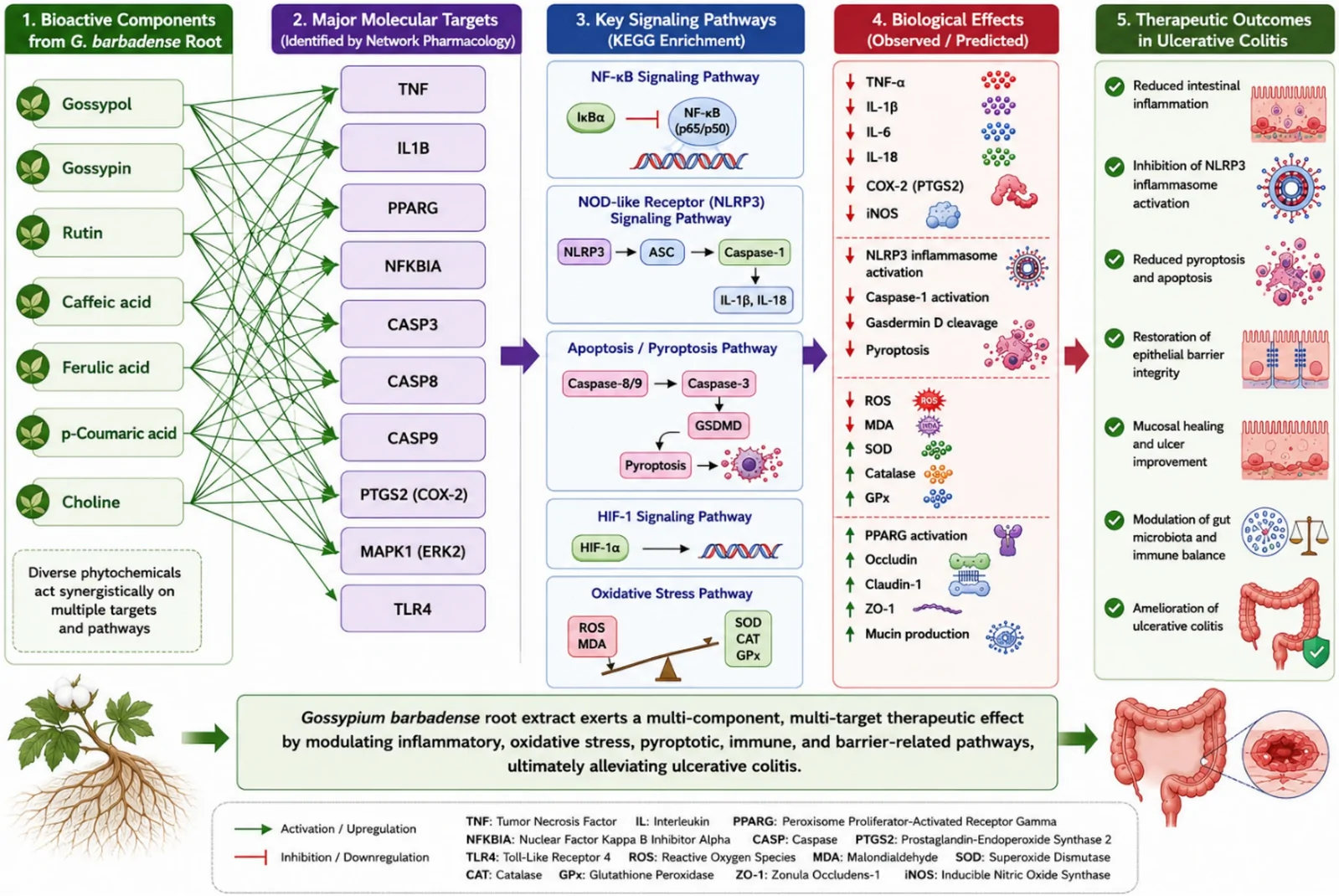
Proposed molecular mechanism underlying the protective effects of *Gossypium barbadense* L. root extract in ulcerative colitis. Major phytochemicals identified in *G. barbadense* root act synergistically on multiple molecular targets, including TNF, IL1B, PPARG, NFKBIA, CASP3, CASP8, CASP9, PTGS2, MAPK1, and TLR4. Regulation of the NF-κB, NOD-like receptor, apoptosis/pyroptosis, HIF-1, and oxidative stress pathways suppresses intestinal inflammation, oxidative injury, and epithelial cell death while enhancing antioxidant defenses, intestinal barrier integrity, mucosal repair, and immune homeostasis, leading to attenuation of ulcerative colitis

The identified target distribution suggests that *G. barbadense* constituents may influence multiple stages of inflammatory signaling, including cytokine production, inflammasome activation, and downstream inflammatory responses (Fig. 14).

### Proposed multi-target therapeutic mechanism

Integration of compound-target interactions with pathway enrichment analysis enabled construction of a proposed mechanistic model for the therapeutic activity of *G. barbadense* in ulcerative colitis (Table 4).

**Table 4:** Proposed multi-target mechanism of Gossypium barbadense in ulcerative colitis

Functional Phase	Key Targets (Pink in KEGG)	Biological Outcome
Priming Phase	NF-kappaB	Inhibition of pro-inflammatory cytokine transcription
Activation Phase	Caspase-1	Suppression of the NLRP3 inflammasome assembly
Execution Phase	IL-1beta	Prevention of pyroptotic cell death and tissue damage
Homeostasis	PPARG	Promotion of mucosal barrier

The analysis suggests that the extract simultaneously modulates several complementary biological processes, including suppression of NFK-β mediated inflammatory signaling, regulation of inflammasome-associated pathways, attenuation of cytokine production, and promotion of epithelial homeostasis through PPARG-mediated signaling. Collectively, these findings support an integrative, network-level mechanism of action characteristic of botanical medicines.

## Discussion

The present network pharmacology analysis provides a comprehensive molecular framework supporting the therapeutic potential of *Gossypium barbadense* root in ulcerative colitis. Unlike conventional pharmacological agents that typically target a single protein or signaling pathway, *G. barbadense* appears to exert its biological effects through coordinated modulation of multiple interconnected inflammatory networks.

One of the most notable findings of this review is the identification of TNF-α as the principal hub gene within the protein interaction network. TNF-α is widely recognized as a master regulator of intestinal inflammation and represents the therapeutic target of several clinically approved biologics for inflammatory bowel disease (Katsanos and Papadakis 2017; Silva et al. 2019). The central position of TNF-α within the predicted interaction network therefore provides a strong molecular rationale for the anti-inflammatory activity of *G. barbadense* reported in experimental models (Alharbi et al. 2025).

In addition to TNF-α, the enrichment of NF-κB, IL-1β, and CASP3 further suggests that *G. barbadense* may influence multiple stages of the inflammatory cascade. NFKB regulates transcription of numerous pro-inflammatory cytokines, whereas IL-1β serves as a major downstream mediator of inflammasome activation (Alsawak et al. 2023b). Caspase-associated signaling is closely linked to inflammatory cell death pathways that contribute to epithelial injury during ulcerative colitis (Amalraj et al. 2017). Although these computational findings do not establish direct inhibition of pyroptosis, they support the hypothesis that *G. barbadense* phytochemicals may modulate inflammasome-related signaling pathways, warranting further experimental validation.

Another important observation is the identification of PPARG as a secondary hub gene. Beyond its established anti-inflammatory functions, PPARG contributes to maintenance of intestinal epithelial barrier integrity and regulation of immune homeostasis (Noori et al. 2024). Simultaneous modulation of TNFα-mediated inflammation and PPARG-dependent barrier protection suggests that *G. barbadense* may exert both anti-inflammatory and tissue-protective effects, representing an advantage over therapies directed toward a single molecular target.

The predicted interaction between choline and muscarinic acetylcholine receptors, particularly CHRM3, also introduces the possibility that cholinergic signaling may contribute to the therapeutic profile of *G. barbadense*. Since activation of the cholinergic anti-inflammatory pathway has been implicated in regulation of intestinal immune responses (Kanauchi et al. 2022), these findings raise the possibility that the extract may influence neuroimmune communication within the gut. However, this hypothesis remains computational and requires validation through functional studies.

Overall, the present network pharmacology analysis demonstrates that *Gossypium barbadense* root possesses a characteristic multi-component, multi-target mode of action, whereby diverse phytochemicals collectively regulate inflammatory cytokines, oxidative stress, epithelial barrier integrity, immune signaling, and pyroptosis-related pathways. These findings provide a molecular basis for the traditional use of *G. barbadense* in inflammatory disorders and support its potential as a complementary therapeutic approach for ulcerative colitis. Nevertheless, network pharmacology remains a predictive computational strategy, and the identified compound–target interactions require further validation through molecular docking, transcriptomic and proteomic analyses, as well as rigorous in vitro, in vivo, and clinical studies.

### Future perspectives and translational challenges

To systematically bridge these in silico predictions with clinical utility, future research must address four core operational pillars:

1. *Botanical Authentication & Extraction Optimization*: Ensure rigorous authentication of the plant material and optimize the extraction process using standardized solvent systems to guarantee reproducible yields of the target bioactive fractions.
2. *Validation & Mechanism*: Move beyond predictive models by deploying molecular docking alongside transcriptomic profiling to confirm exact compound-target interfaces during experimental colitis.
3. *Safety & Standardization*: Develop reproducible extraction baselines to control natural phytochemical variance and carefully monitor dose-dependent toxicities associated with specific native markers like gossypol.
4. *Bioavailability Enhancement*: Implement targeted pharmaceutical design, such as nano-formulations or advanced encapsulation delivery systems, to ensure the active fractions survive gastrointestinal transit and achieve optimal therapeutic concentrations directly at the inflamed colonic mucosa.

## Conclusion

*Gossypium barbadense* L. root is a promising phytotherapeutic candidate for ulcerative colitis owing to its rich composition of flavonoids, phenolic compounds, terpenoids, alkaloids, tannins, saponins, and glycosides. These bioactive constituents collectively exert antioxidant, anti-inflammatory, immunomodulatory, and mucosal protective effects by reducing oxidative stress, suppressing pro-inflammatory mediators, preserving intestinal barrier integrity, and promoting mucosal healing.

Network pharmacology further supports these findings by identifying TNF-α, PPARG, IL1-β, NFKBIA, and Caspase-3 as key molecular targets and highlighting the involvement of the NFK-β, NOD-like receptor, and apoptosis/pyroptosis signaling pathways. Together, these findings suggest that *G. barbadense* exerts therapeutic effects through a multi-component, multi-target mechanism, making it a promising complementary strategy for ulcerative colitis management.

Nevertheless, network pharmacology is inherently predictive, and the proposed compound target interactions require validation through molecular docking, transcriptomic, proteomic, and well-designed experimental and clinical studies. Such investigations are essential to confirm the molecular mechanisms, establish safety and efficacy, and facilitate the translation of *G. barbadense* root into evidence-based phytotherapy for ulcerative colitis.

## Data Availability

All data supporting the findings of this study are available within the paper and its Supplementary Information.

## Abbreviations

5-ASA: 5-Aminosalicylic acid
AKBA: Acetyl-keto-β-boswellic acid
APCs: Antigen-presenting cells
BAs: Boswellic acids
BCL-2: B-cell Lymphoma 2
BH-3: BCL-2 homology domain
CASP-3: Caspase-3
CD: Crohn’s disease
CMV: Cytomegalovirus
COX-2: Cyclooxygenase-2
CT: Computed tomography
GPx: Glutathione peroxidase
HPLC: High performance liquid chromatography
IBD: Inflammatory bowel disease
IL: Interleukin
iNOS: Inducible nitric oxide synthase
KBA: Keto-β-boswellic acid
LOX: Lipoxygenase
LTB4: Leukotriene B4
MAPK: Mitogen-activated protein kinase
MCJ: Methylation-controlled-J-protein
MDA: Malondialdehyde
MRI: Magnetic resonance imaging
mROS: Mitochondrial reactive oxygen species
NF-κB: Nuclear factor-kappa B
NO: Nitric oxide
NSAIDs: Nonsteroidal anti-inflammatory drugs
PGC-1α: Peroxisome proliferator-activated receptor-γ coactivator 1α
RNS: Reactive nitrogen species
ROS: Reactive oxygen species
SCFAs: Short chain fatty acids
SOD: Superoxide dismutase
STAT3: Signal transducer and activator of transcription 3
Th: T helper cell
TNF-α: Tumer necrosis factor-alpha
Tregs: Regulatory T cells
UC: Ulcerative colitis
